# Non-invasive glycemic control leveraging ultrasound dose window activation of MscL in diabetic mice

**DOI:** 10.1016/j.isci.2026.117962

**Published:** 2026-10-19

**Authors:** Xinyi Zhang, Jun Zhan, Wenjin Mao, Xinbin Han, Yu Chen, Jianghao Chen, Yan Wang

**Affiliations:** 1 State Key Laboratory of Ultrasound in Medicine and Engineering, College of Biomedical Engineering, Chongqing Medical University, Chongqing 400016, China

**Keywords:** sonogenetics, mechanosensitive ion channels, ultrasound, β cell, insulin secretion, type 2 diabetes

## Abstract

Type 2 diabetes is characterized by impaired glucose homeostasis and progressive pancreatic β cell dysfunction, highlighting the need for externally controllable strategies for metabolic regulation. Here, we combined low-intensity pulsed ultrasound (LIPUS) with the mechanosensitive channel MscL-G22S to establish a tunable sonogenetic system for glucose regulation. Graded ultrasound stimulation was evaluated in MscL-G22S-expressing β cells and diabetic db/db mice to define an effective acoustic dose range. Within an intermediate dose window, LIPUS increased intracellular Ca^2+^ levels and glucose-stimulated insulin secretion *in vitro* and improved glycemic control, insulin sensitivity, islet morphology, and systemic metabolic profiles *in vivo*. Serum metabolomics further identified metabolic pathway alterations associated with combined LIPUS and MscL-G22S activation. These findings establish a quantitative framework for relating ultrasound dose to sonogenetic metabolic responses and support further investigation of ultrasound-controlled regulation of glucose homeostasis.

## Highlights

• LIPUS activates MscL-G22S to non-invasively regulate glucose homeostasis in diabetic mice
• An optimal acoustic dose window (3.5–5.9 a.u.) maximizes glycemic control in db/db mice
• MscL-G22S enables ultrasound-controlled Ca^2+^ influx and insulin secretion in beta cells
• Serum metabolomics reveals pathway-level metabolic remodeling by sonogenetic stimulation

## Graphical abstract

**Figure:**
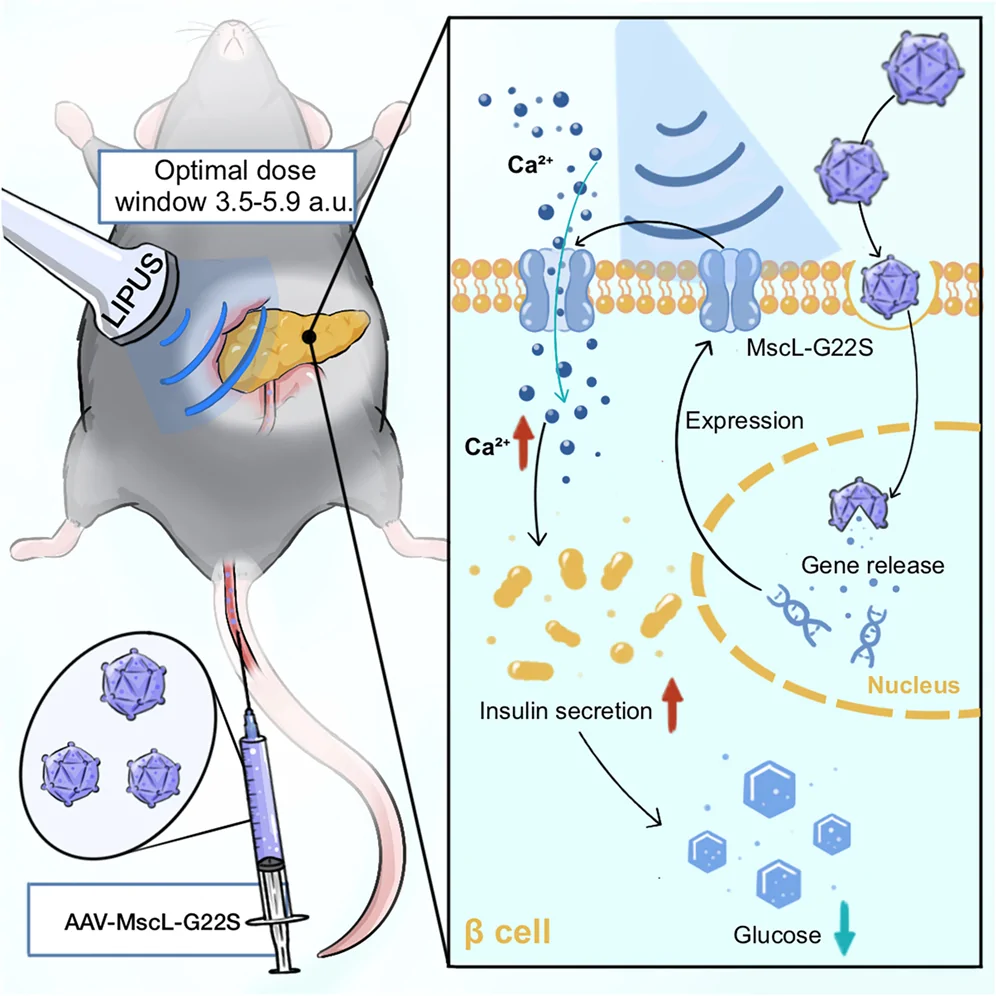

## Teaser

Molecular biology; Functional aspects of cell biology

## Introduction

Type 2 diabetes mellitus (T2DM) is one of the most prevalent metabolic disorders worldwide, and its prevalence continues to rise at an alarming rate. According to the 11^th^ edition of the International Diabetes Federation (IDF) Diabetes Atlas, 589 million people aged 20–79 years lived with diabetes globally in 2024, and this number is expected to rise to 853 million by 2050, with T2DM accounting for over 90% of all cases.^1^ The progressive loss of pancreatic β cell function combined with insulin resistance disrupts systemic glucose homeostasis and drives widespread metabolic complications. Although lifestyle intervention and pharmacological treatments remain the cornerstone of therapy, their long-term effectiveness is frequently constrained by adverse effects, limited adherence, and the inability to dynamically regulate insulin secretion with spatial and temporal precision.^2^^,^^3^ These limitations highlight a fundamental need for strategies capable of remotely controlling metabolic function in a tunable, non-invasive manner.

Low-intensity ultrasound has emerged as a promising modality for non-invasive biological modulation. Previous studies have shown that therapeutic ultrasound can stimulate insulin secretion and alleviate chronic inflammation associated with T2DM, primarily through mechanical perturbation of cellular membranes.^4^^,^^5^^,^^6^^,^^7^ However, metabolic outcomes induced by ultrasound vary substantially across studies and stimulation conditions. A central unresolved challenge is the lack of a quantitative framework linking acoustic energy input to metabolic output. Without a clearly defined and reproducible stimulation window, ultrasound-based metabolic modulation remains empirically driven and difficult to standardize, limiting its translational reliability. Part of this variability can be attributed to confounding contributions from endogenous mechanosensitive pathways in β cells, most notably Piezo1, which mediates Ca^2+^ influx, electrical activity, and insulin secretion in response to mechanical and glucose-derived stimuli. While Piezo1 is essential for physiological glucose-induced insulin secretion; its expression, membrane localization, and activation thresholds are highly heterogeneous. These properties are dynamically regulated by metabolic state, extracellular matrix stiffness, and disease conditions.^8^^,^^9^ For example, Piezo1 undergoes nuclear translocation and functional inactivation under chronic high glucose, a hallmark of type 2 diabetes, further undermining consistent responsiveness.^8^^,^^10^ Such inherent variability mirrors limitations of other endogenous metabolic ion channels (e.g., transient receptor potential vanilloid 1 [TRPV1] and transient receptor potential ankyrin 1 [TRPA1]), which exhibit divergent expression and activation profiles that preclude reliable and reproducible modulation by external stimuli.^11^^,^^12^ Critically, native mechanosensitive pathways may introduce non-specific confounding signals and off-target effects, making it difficult to unambiguously attribute ultrasound responses to direct channel gating.^13^^,^^14^ These limitations highlight the need for an exogenous mechanosensitive system with well-defined activation properties to achieve precise and reproducible ultrasound-mediated modulation.

Mechanosensitive channel-based strategies have been increasingly validated in mammalian systems, ranging from Piezo1-mediated ultrasound neuromodulation to targeted sonogenetic control of defined cell populations. Among these, the bacterial mechanosensitive channel MscL and its gain-of-function mutant MscL-G22S offer distinct advantages: compared with endogenous channels such as Piezo1 or TRPV4, MscL-G22S is primarily activated by membrane tension, exhibits a relatively well-defined activation threshold, and shows limited coupling to endogenous signaling pathways, enabling more consistent and tunable responses to low-intensity ultrasound in sonogenetic applications.^15^^,^^16^^,^^17^ Sonogenetics provides a potential solution by coupling mechanosensitive ion channels (MSCs) with ultrasound stimulation to create a programmable interface between physical energy and cellular activity.^18^^,^^19^ Among MSCs, the bacterial large-conductance mechanosensitive channel MscL has attracted considerable interest because of its well-characterized gating mechanism, large pore size, and direct responsiveness to membrane tension.^20^^,^^21^^,^^22^ Importantly, the gain-of-function mutant MscL-G22S exhibits enhanced pressure sensitivity, allowing controlled channel activation in response to mechanical stimuli such as ultrasound.^23^^,^^24^^,^^25^^,^^26^ These properties position MscL-G22S as a biological actuator capable of translating defined acoustic inputs into predictable intracellular signaling events.

Building on this concept, we hypothesized that systematic optimization of low-intensity pulsed ultrasound (LIPUS) parameters in combination with pancreatic MscL-G22S expression could establish a quantifiable acoustic dose window for remote glycemic regulation. Using adeno-associated virus (AAV)-mediated delivery and graded acoustic stimulation, we mapped the relationship between ultrasound dose and sonogenetic responses *in vitro*, as well as the resultant metabolic regulation in diabetic db/db mice. We confirmed biologically effective responses within a defined acoustic range. *In vitro*, ultrasound activation of MscL-G22S induced controlled calcium influx and enhanced insulin secretion in β-cells. *In vivo*, acoustic stimulation within this window improved glycemic control, preserved islet architecture, and promoted systemic metabolic remodeling without detectable toxicity, with β-cells serving as one of the potential participants in this regulatory network.

By explicitly defining a reproducible coupling between acoustic stimulation intensity and systemic metabolic responses, this study advances mechanogenetics from a qualitative stimulation approach toward a quantitative and tunable framework for non-invasive metabolic regulation. The identification of a defined acoustic response window provides a rational basis for future sonogenetic modulation of glucose homeostasis and highlights the potential of ultrasound-controlled metabolic regulation as a therapeutic strategy for T2DM.

## Results

### Establishment of a diabetic mouse model and pancreatic expression of mechanosensitive MscL-G22S

As illustrated in the experimental timeline and conceptual schematic (Figure 1A), systemic AAV delivery achieved robust pancreatic expression of MscL-G22S. EGFP fluorescence colocalized with insulin-positive β cells, confirming successful targeting (Figure 1B), which was further supported by significantly increased pancreatic MscL mRNA levels measured by RT-qPCR (Figure 1C); overall transduction efficiency and distribution are shown in Figure S1. Male C57BKS-db/db mice displayed elevated fasting blood glucose and impaired glucose tolerance compared with age-matched db/m controls before ultrasound intervention, validating the diabetic model (Figures 1D and 1E).

**Figure 1:**
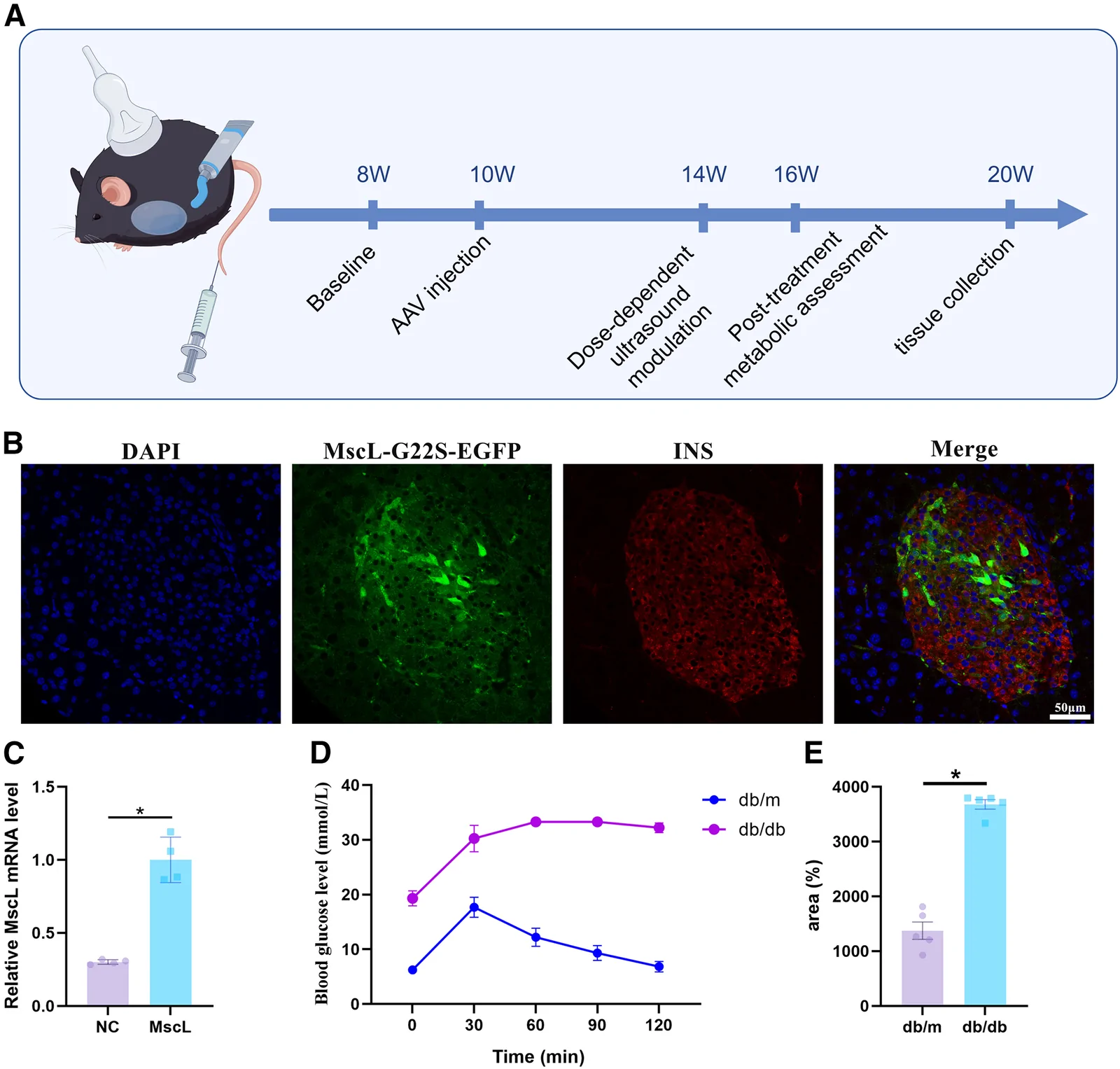
Verification of AAV-mediated MscL-G22S expression and baseline diabetic characterization in db/db mice (A) Experimental timeline and treatment scheme. (B) Immunofluorescence staining of pancreatic islets showing DAPI (blue), MscL-G22S-EGFP (green), insulin (red), and merged images, confirming successful AAV transduction. Scale bars, 50 μm. (C) RT-qPCR measurement of pancreatic MscL mRNA expression. (D) Baseline glucose tolerance test (GTT) curves in db/m and db/db mice. (E) Quantification of GTT area under the curve (AUC), demonstrating impaired glucose tolerance in db/db mice. Data are mean ± SD; *n* = 4 mice per group (C) and *n* = 5 mice per group (D and E). Statistical significance was determined by unpaired two-tailed Student’s *t* test (C and E) and by a mixed-effects model (REML) with the Geisser-Greenhouse correction followed by Tukey’s post hoc test (D). ∗*p* < 0.05. Exact test statistics (*F*/*t* values), degrees of freedom, and exact *p* values are provided in Table S2.

### Dose-dependent *in vivo* glycemic regulation reveals an optimal acoustic window

Baseline blood glucose levels were comparable across all experimental groups prior to LIPUS treatment. After the first 10-day round of LIPUS exposure (Table 1), ultrasound-treated mice showed modest reductions in blood glucose relative to mock controls, with the largest decreases observed in the intermediate-dose groups (G2–G4), although these differences did not reach statistical significance at this stage (Figures 2A and 2B). Analysis of the absolute change in blood glucose (Δglucose) before and after treatment suggested a non-linear dose-response relationship: Locally estimated scatterplot smoothing (LOESS) fitting revealed an inverted U-shaped trend, with maximal glucose reduction occurring at intermediate doses (Figure 2C). A second round of LIPUS treatment produced clearer dose-dependent effects. Within-group comparisons showed significant glucose reductions in the G2, G3, and G5 groups, and Δglucose during the second round differed significantly across dose groups, with the most pronounced reductions versus mock observed in the G3 and G4 groups (Figures 2D and 2E). Endpoint dose-response analysis confirmed maximal glycemic regulation within the intermediate-dose range, establishing that effective *in vivo* metabolic control is confined to a discrete range of ultrasound parameters (Figure 2F). Longitudinal monitoring of metabolic parameters over 12 weeks (8–20 weeks of age) further supported these findings. Random blood glucose (Figure 2G) remained lower in the G2–G4 groups compared with mock controls, with significant differences emerging from 14 to 16 weeks onward. Fasting blood glucose (Figure 2H) showed a similar pattern, indicating effective mitigation of hyperglycemia in the G2–G4 groups. Body weight (Figure 2I) increased progressively across all db/db groups, with no significant differences between treatment groups, suggesting that LIPUS did not affect overall growth. Daily water intake (Figure 2J) was reduced in the G2–G4 groups, consistent with alleviation of polydipsia and improved metabolic status.

**Table 1:** Experimental groups and ultrasound parameter settings for the *in vivo* dose-dependent study

Group	Ultrasound dose (a.u.)	Average intensity (mW/cm^2^)	Duty cycle (%)	Frequency (MHz)	Treatment days	Irradiation time (min/day)
Group 1	2.352	400	20	0.36	10	10
Group 2	3.528	600	20	0.36	10	10
Group 3	4.704	400	40	0.36	10	10
Group 4	5.880	1,000	20	0.36	10	10
Group 5	7.056	800	30	0.36	10	10

**Figure 2:**
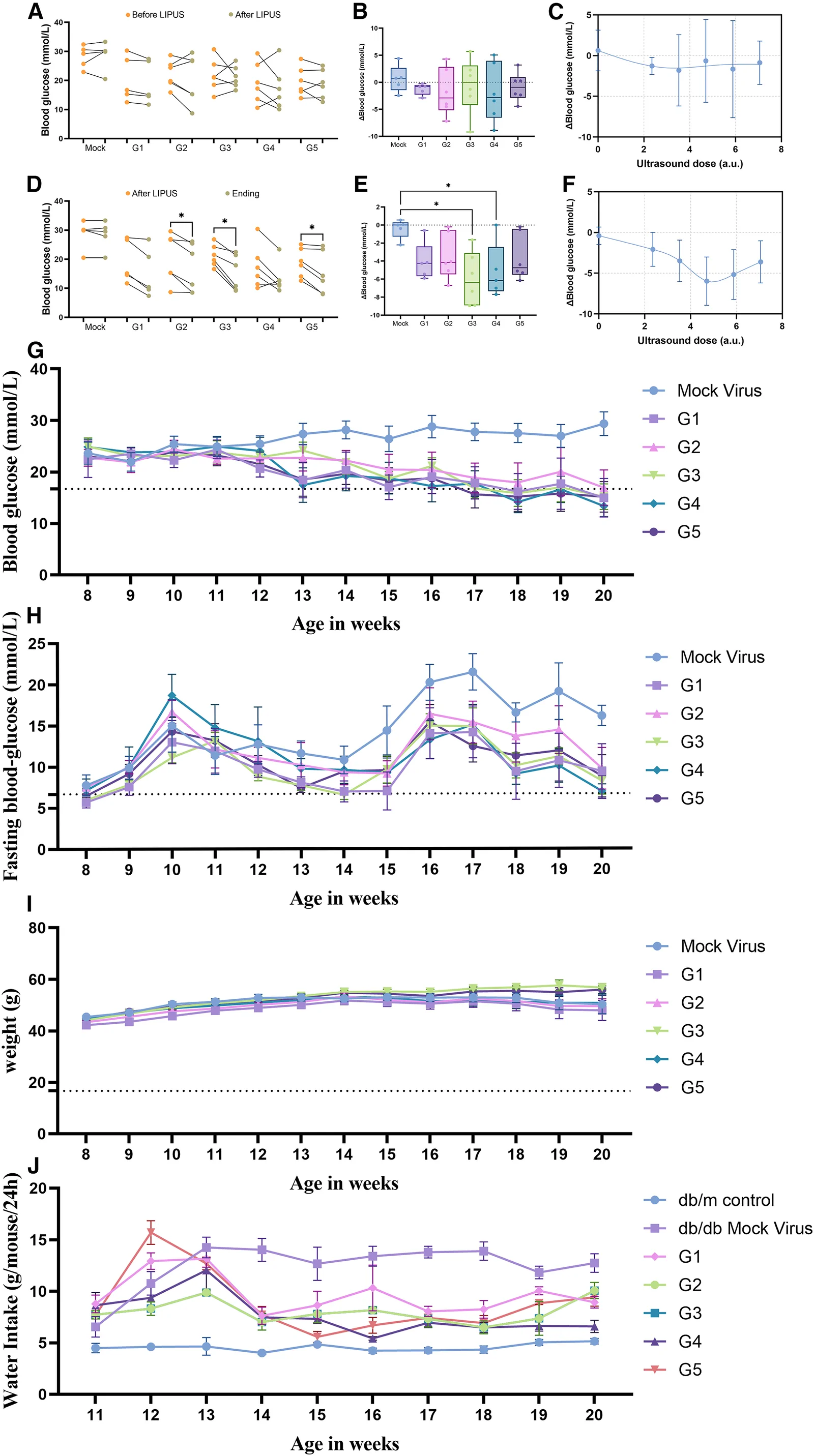
Dose-dependent blood glucose reduction induced by LIPUS in MscL-G22S-expressing db/db mice (A) Random blood glucose levels before and after a single round of LIPUS stimulation across different ultrasound dose groups (G1–G5). Mock: no LIPUS treatment. (B) Boxplots showing the change in blood glucose (Δblood glucose) before versus after LIPUS in each dose group. (C) Dose-response relationship between ultrasound dose and Δblood glucose after the first treatment session; LOESS fitting demonstrates a non-linear, inverted U-shaped relationship with maximal glucose reduction at intermediate doses. (D) Random blood glucose levels immediately after LIPUS and at the end of the second treatment cycle. (E) Boxplots of Δblood glucose during the second treatment round, illustrating dose-dependent differences among groups. (F) Dose-response curve of the second LIPUS session, showing maximal glucose reduction at moderate ultrasound doses (approximately 3.5–5.9 a.u., groups G2–G4), indicating a clear dose-dependent activation of MscL-G22S *in vivo*. (G) Random blood glucose levels measured weekly from 8 to 20 weeks of age in mice receiving mock treatment or different ultrasound dose conditions (G1–G5). (H) Fasting blood glucose levels monitored over the same experimental period. (I) Body weight changes recorded longitudinally during the study. (J) Daily water intake measured between 11 and 20 weeks of age in db/m control mice, db/db mock mice, and ultrasound-treated groups (G1–G5). Data are mean ± SD; *n* = 5–6 mice per group (A–I) and *n* = 4 mice per group (J). Before-versus-after comparisons within each group were analyzed using two-tailed Wilcoxon matched-pairs signed-rank tests (A and D); differences in Δblood glucose across dose groups were analyzed by one-way ANOVA with Tukey’s post hoc test (B) or one-way ANOVA with Dunnett’s post hoc test versus the mock group (E); longitudinal data (G–J) were analyzed by two-way repeated-measures ANOVA with Geisser-Greenhouse correction followed by Dunnett’s post hoc test versus the mock group within each week. ∗*p* < 0.05 was considered statistically significant. Exact test statistics (*F* values), degrees of freedom, and exact *p* values are provided in Table S2.

Collectively, these results demonstrate that LIPUS-mediated glycemic regulation in db/db mice is non-linear and dose-dependent, with an optimal therapeutic window observed in the G2–G4 groups. This effect is sustained across multiple metabolic parameters without compromising general health indicators such as body weight.

### Dose-dependent LIPUS improves insulin sensitivity and metabolic profiles in db/db mice

Following LIPUS treatment, fasting insulin levels tended to decrease in ultrasound-treated groups, accompanied by a significant reduction in the homeostatic model assessment for insulin resistance (HOMA-IR). The greatest improvement was observed in the moderate-dose groups (G3–G4) at the experimental endpoint (4 weeks after completion of LIPUS treatment) (Figures 3A–3D), indicating a sustained metabolic effect. LIPUS also promoted favorable systemic lipid changes: high-density lipoprotein cholesterol (HDL-C) levels increased significantly in G2–G4, whereas low-density lipoprotein cholesterol (LDL-C), total cholesterol, and triglycerides were moderately reduced, with the most pronounced effects in the moderate-dose groups (Figures 3E–3H). Long-term glycemic exposure was assessed by glycosylated serum protein (GSP) levels, which represent a time-integrated index of average glycemic exposure over the preceding 2–3 weeks rather than a single time-point measurement. Consistent with the longitudinal blood glucose profiles, ultrasound-treated groups exhibited lower GSP levels compared with the mock group, reflecting reduced cumulative glycemic burden during late-stage disease progression (Figure 3I). In contrast, the epididymal white adipose tissue (eWAT) to body weight ratio remained unchanged across groups (Figure 3J), indicating that metabolic improvements were independent of adipose mass. Glucose tolerance test (GTT) and insulin tolerance test (ITT) were evaluated at the end of the study and are presented in Figure S2, confirming enhanced glucose handling in the moderate-dose groups.

**Figure 3:**
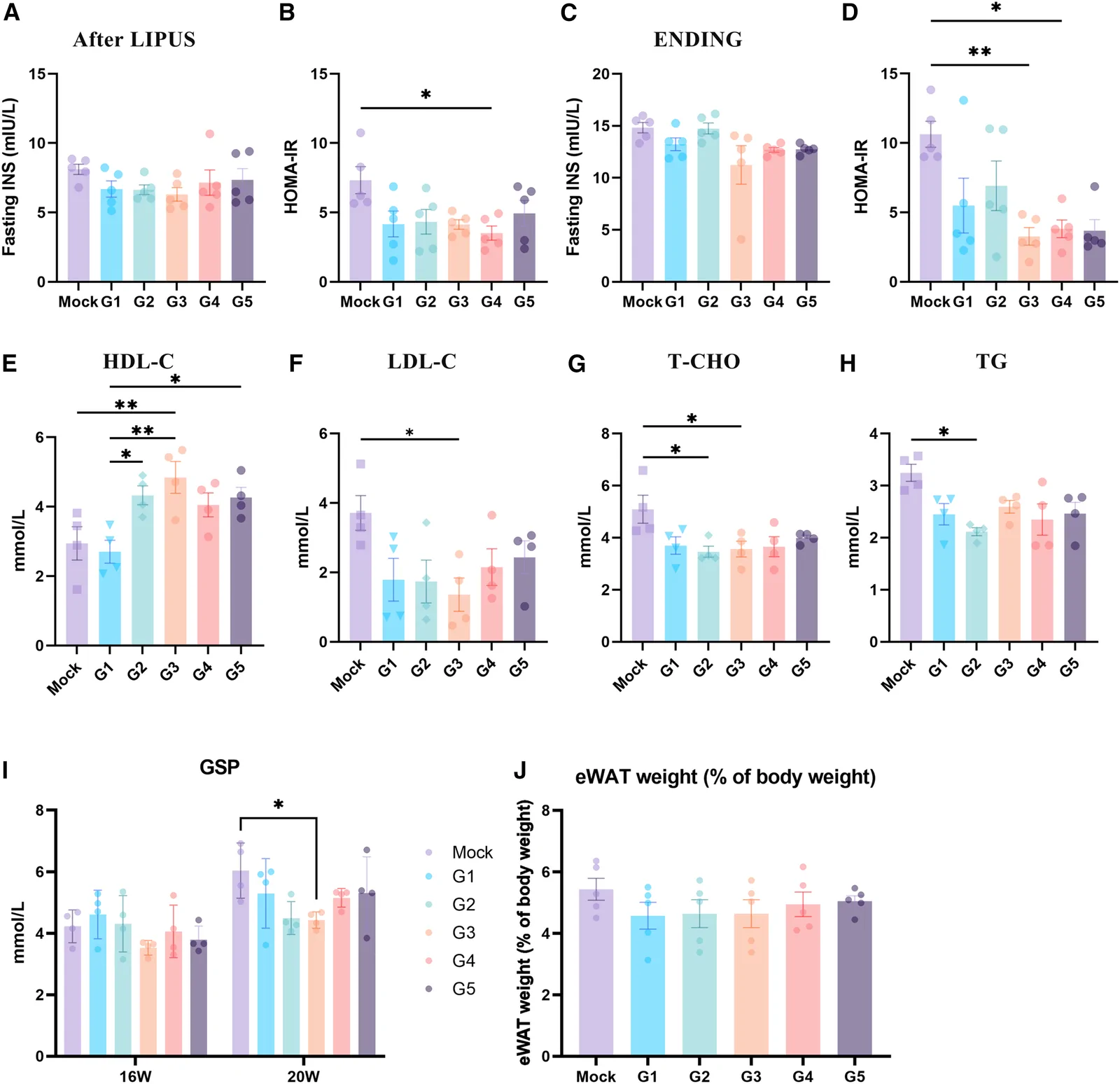
Metabolic improvements induced by dose-dependent LIPUS stimulation in db/db mice (A and B) After LIPUS treatment, fasting insulin levels (A) and HOMA-IR index (B) in different ultrasound-dose groups (mock, G1–G5). LIPUS at moderate doses significantly reduced insulin resistance. (C and D) Fasting insulin (C) and HOMA-IR (D) measured at the experimental endpoint (4 weeks after completion of LIPUS treatment). (E) HDL-C was significantly increased in the G2–G4 groups. (F–H) LDL-C (F), total cholesterol (TC, G), and triglycerides (TG, H) were moderately reduced, indicating improved systemic lipid metabolism following LIPUS stimulation. (I) Glycosylated serum protein (GSP) levels measured at 16 and 20 weeks of age; GSP represents cumulative glycemic exposure over the preceding 2–3 weeks. (J) Ratio of epididymal white adipose tissue (eWAT) to body weight; LIPUS stimulation did not significantly alter adipose tissue mass across groups. Data are mean ± SD; *n* = 5 mice per group (A–D and J) and *n* = 4 mice per group (E–I). Statistical significance was determined using one-way ANOVA with Dunnett’s post hoc multiple-comparisons test versus the mock group. ∗*p* < 0.05, ∗∗*p* < 0.01, ∗∗∗*p* < 0.001, ∗∗∗∗*p* < 0.0001; ns, not significant. Exact test statistics (*F* values), degrees of freedom, and exact *p* values are provided in Table S2.

### Conventional LIPUS parameters show limited glycemic improvement prior to dose optimization

Before systematic acoustic dose optimization, a conventional LIPUS parameter protocol previously used in our group (1.176 arbitrary units units or a.u.) was applied to db/db mice expressing pancreatic MscL-G22S. Longitudinal monitoring of random blood glucose from 8 to 20 weeks of age revealed that LIPUS combined with MscL-G22S elicited a modest reduction in glycemic levels compared with Mock db/db controls, although the overall effect remained limited and variable (Figure 4A). Body weight trajectories showed no significant differences among treatment groups during the intervention period (Figure 4B). GTTs demonstrated partial improvement in glucose clearance following LIPUS treatment, particularly in the MscL-overexpressing group (Figure 4C). Consistently, fasting blood glucose levels were reduced after the 10-day LIPUS intervention (Figure 4D), although paired analysis of individual mice revealed considerable inter-individual variability in treatment response (Figure 4E). Histological evaluation by hematoxylin-eosin (H&E) staining showed partial preservation of islet architecture in LIPUS-treated MscL-G22S mice; however, islet hypertrophy and structural disorganization were still evident compared with non-diabetic controls (Figure 4F). Immunofluorescence staining further demonstrated improved islet cellular composition, with enhanced insulin-positive β cell area and relatively reduced glucagon-positive α cell distribution (Figure 4G), leading to a decreased α/β cell ratio in the MscL-overexpressing group (Figure 4H). H&E staining of major organs revealed no obvious pathological alterations, with detailed results provided in Figure S3.

**Figure 4:**
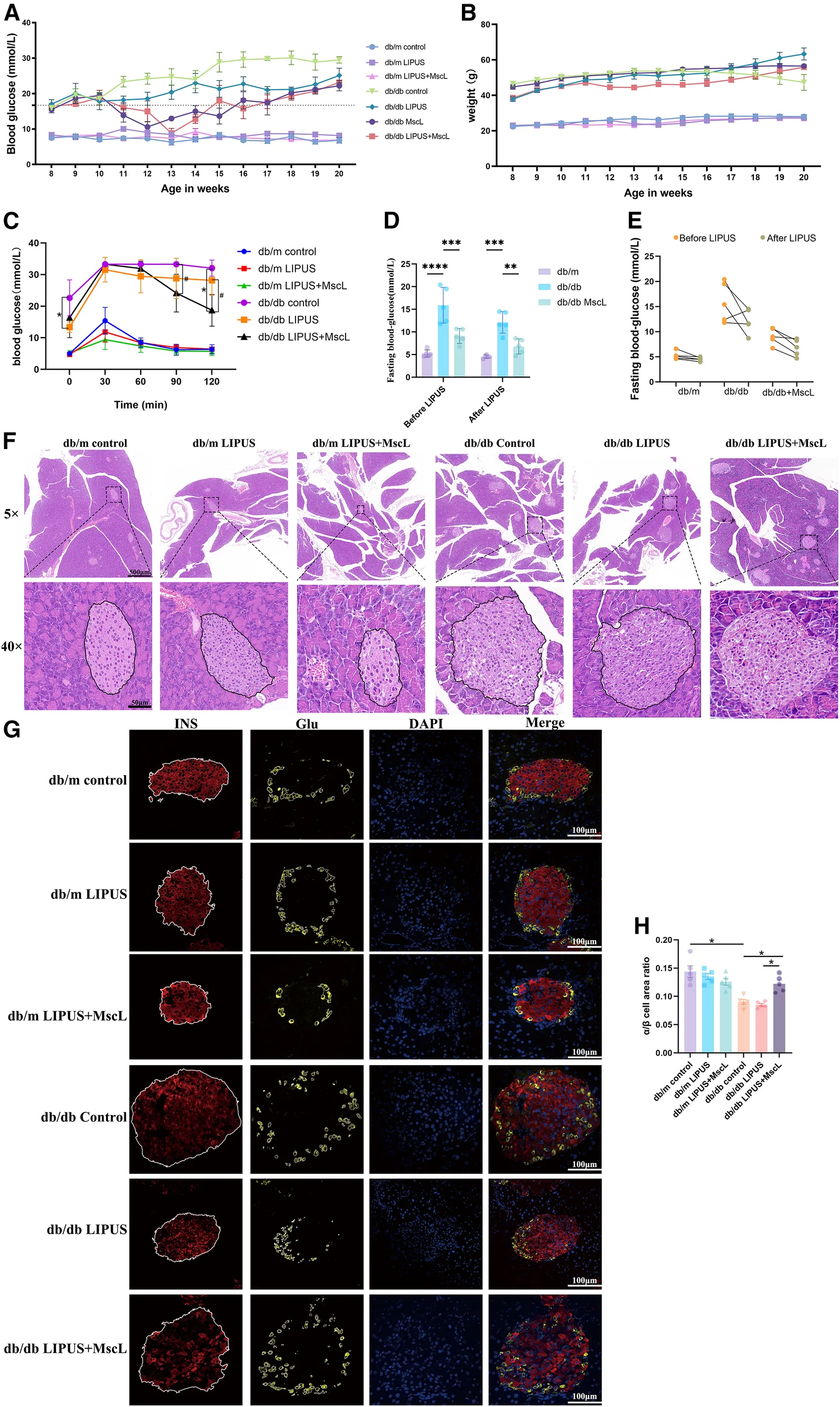
LIPUS combined with MscL-G22S improves glycemic control and preserves islet architecture in db/db mice (A) Longitudinal monitoring of random blood glucose levels from 8 to 20 weeks of age in db/m and db/db mice receiving mock, LIPUS, or LIPUS + MscL treatment. (B) Body weight curves during the treatment period. (C) Glucose tolerance test (GTT). (D) Fasting blood glucose levels before and after the 10-day LIPUS intervention. (E) Paired comparison of individual fasting blood glucose values before and after LIPUS treatment in each group. (F) Representative H&E staining of pancreatic islets showing morphological changes across treatment groups (upper images, 5× objective; lower images, 40× objective). Scale bars, 500 μm (upper) and 50 μm (lower). (G) Immunofluorescence staining of insulin (INS), glucagon (Glu), and nuclei (DAPI) in pancreatic islets. Scale bars, 100 μm. (H) Quantification of the α/β cell ratio based on the relative immunofluorescence-positive area of glucagon-positive (α) and insulin-positive (β) cells within islet regions. Data are mean ± SD; *n* = 4 mice per group (A and B), and *n* = 5 mice per group (C–E and H). Statistical significance was determined by two-way repeated-measures ANOVA with Geisser-Greenhouse correction for longitudinal data, followed by Dunnett’s post hoc test versus the db/db control group (A) or Tukey’s post hoc test (C); between-group differences at each time point in (D) were analyzed by one-way ANOVA with Tukey’s post hoc test, and paired before-versus-after comparisons in (E) were analyzed by paired two-tailed Student’s *t* test; the α/β cell ratio was analyzed by one-way ANOVA with Dunnett’s post hoc test versus the db/db control group (H). ∗*p* < 0.05, ∗∗*p* < 0.01, ∗∗∗*p* < 0.001, ∗∗∗∗*p* < 0.0001; ns, not significant. Exact test statistics (*F*/*t* values), degrees of freedom, and exact *p* values are provided in Table S2.

### MscL-G22S is robustly expressed in 293T and MIN6 cells with membrane localization

To assess MscL-G22S expression and subcellular localization, 293T and MIN6 cells were transfected with MscL-G22S-mCherry or control plasmids. Blank and negative control cells showed minimal background fluorescence, whereas MscL-G22S-transfected 293T cells exhibited strong and uniform mCherry signals (Figure 5A). MIN6 cells displayed a similar expression pattern, with lower apparent fluorescence intensity consistent with intrinsic β cell autofluorescence (Figure 5B). Confocal imaging confirmed predominant membrane-associated localization of MscL-G22S in 293T cells: cells transfected with empty vector showed diffuse cytoplasmic mCherry fluorescence, whereas the MscL-G22S-mCherry fusion protein was clearly enriched at the plasma membrane (Figure 5C). RT-qPCR analysis further showed significantly elevated MscL mRNA levels in transfected MIN6 cells compared with controls (Figure 5D). These results collectively demonstrate efficient expression and correct membrane targeting of MscL-G22S in both cell types.

**Figure 5:**
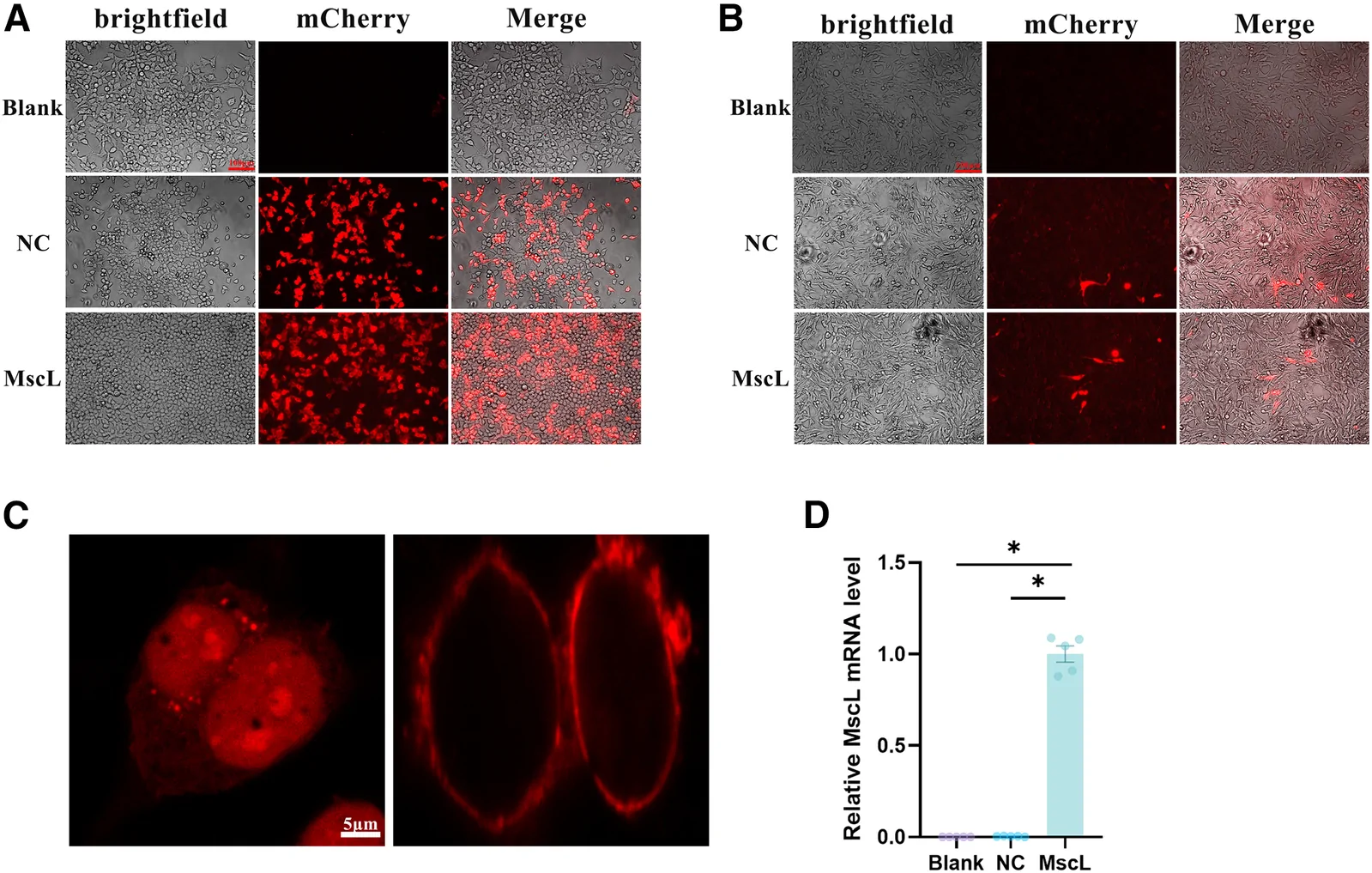
Expression and subcellular localization of MscL-G22S in mammalian cells (A) Representative brightfield, mCherry, and merged images showing reporter expression in 293T cells. Blank and negative control (NC) groups showed minimal fluorescence, whereas cells transfected with the MscL-G22S-mCherry construct displayed robust red fluorescence distributed across the cell population. Scale bars, 100 μm. (B) Expression patterns in MIN6 cells. Compared with blank and NC groups, the MscL-G22S group exhibited detectable mCherry signal, indicating successful plasmid transfection, although overall fluorescence was lower due to the inherent optical properties of MIN6 β-cells. Scale bars, 100 μm. (C) High-magnification confocal images of 293T cells transfected with different constructs. Left image: cells transfected with empty vector expressing mCherry alone. Right image: cells transfected with the MscL-G22S-mCherry fusion construct. Scale bars, 5 μm. (D) RT-qPCR analysis of MIN6 cells confirming significant overexpression of MscL-G22S mRNA in the transfected group compared with the blank and NC (Welch’s one-way ANOVA with Games-Howell post hoc test). Data are mean ± SD; *n* = 5 independent biological replicates per group. ∗*p* < 0.05. Exact test statistics (*F* values), degrees of freedom, and exact *p* values are provided in Table S2.

### MscL-G22S expression is non-toxic and confers ultrasound-responsive enhancement of cell viability

Basal cell viability assays showed that MscL-G22S expression did not affect viability in 293T or MIN6 cells (Figures 6A and 6B). Upon LIPUS stimulation, MscL-G22S-expressing cells exhibited increased viability compared with non-stimulated counterparts, whereas blank and negative control cells showed minimal responses (Figure 6C). Consistent with this result, MscL-G22S-expressing MIN6 cells also showed enhanced viability under the same acoustic stimulation (Figure 6D). Graded acoustic stimulation induced a dose-dependent increase in viability within a defined range, while higher doses failed to further enhance the response, showing a plateau or slight decline (Figure 6E). These results indicate that MscL-G22S expression is well tolerated and confers ultrasound responsiveness at the cellular level, with viability changes following a non-linear dependence on acoustic dose.

**Figure 6:**
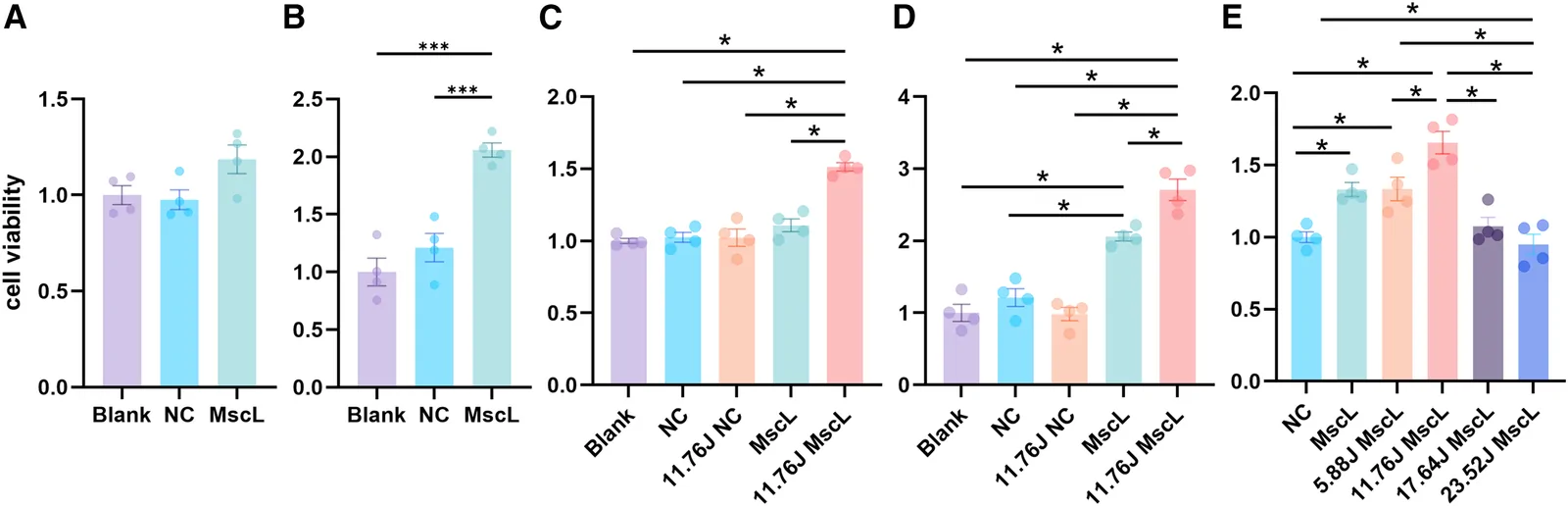
Dose-dependent enhancement of cell viability by ultrasound activation of MscL-G22S (A) Basal cell viability of 293T cells transfected with MscL-G22S or negative control (NC) plasmids, showing no significant cytotoxicity of MscL expression. (B) Basal viability of MIN6 β-cells, confirming that MscL-G22S overexpression does not impair cellular health. (C) Cell viability of 293T cells following ultrasound stimulation (11.76 a.u.). MscL-expressing cells exhibited significantly enhanced viability compared with NC and blank groups. (D) Cell viability of MIN6 cells following ultrasound stimulation (11.76 a.u.). MscL-expressing cells exhibited significantly enhanced viability compared with NC and blank groups. (E) Integrated dose-response analysis in MIN6 cells showing viability changes across multiple ultrasound doses (5.88–23.52 a.u.). A non-linear effect was observed, with viability peaking at 11.76 a.u. and declining at the highest dose (23.52 a.u.). Data are mean ± SD; *n* = 4 independent biological replicates per group. Statistical analysis was performed using one-way ANOVA with Tukey’s post hoc test. ∗*p* < 0.05, ∗∗*p* < 0.01, ∗∗∗*p* < 0.001, ∗∗∗∗*p* < 0.0001; ns, not significant. Exact test statistics (*F* values), degrees of freedom, and exact *p* values are provided in Table S2.

### *In vitro* Ca^2+^ influx and insulin secretion exhibit a non-linear response to acoustic stimulation

In MIN6 β-cells expressing MscL-G22S, glucose-stimulated insulin secretion (GSIS) was enhanced following ultrasound stimulation (Table 2) in a dose-dependent manner, with maximal augmentation at the 11.76 a.u. dose. Lower doses produced minimal effects, whereas higher doses failed to further increase insulin secretion (Figures 7A–7C). Consistent with GSIS, ultrasound stimulation induced a non-linear increase in intracellular Ca^2+^ levels: the 11.76 a.u. dose elicited the highest Ca^2+^ influx, while further increases in ultrasound intensity resulted in a plateau or slight decline in Ca^2+^ responses (Figure 7D). Together, these results indicate that *in vitro* cellular responses to ultrasound follow a non-linear dose-response pattern, with optimal Ca^2+^ influx and insulin secretion achieved at the 11.76 a.u. dose.

**Table 2:** Experimental groups and ultrasound parameter settings for the *in vitro* dose-dependent study

Group	Ultrasound dose (a.u.)	Average intensity (mW/cm^2^)	Duty cycle (%)	Frequency (MHz)	Irradiation time (s)
Group 1	5.88	200	20	0.36	30
Group 2	11.76	400	20	0.36	30
Group 3	17.64	600	20	0.36	30
Group 4	23.52	800	20	0.36	30

**Figure 7:**
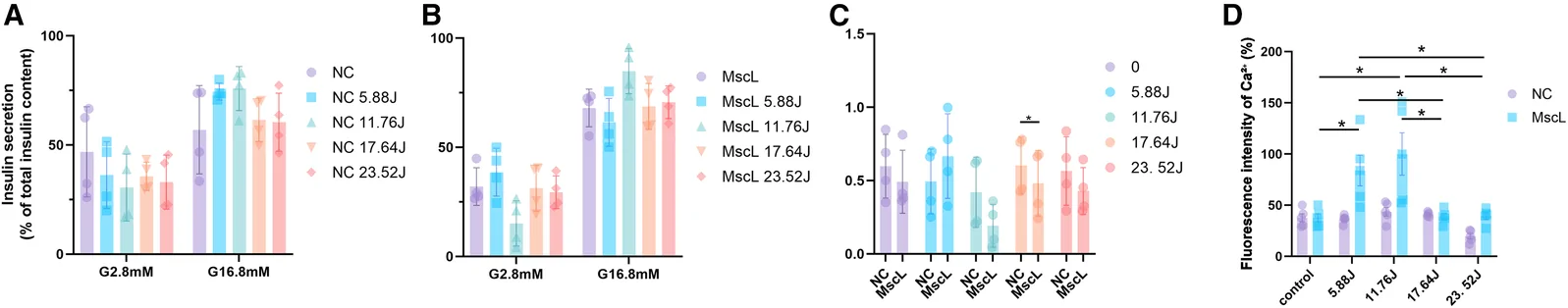
Ultrasound activation of MscL-G22S enhances glucose-stimulated insulin secretion and intracellular Ca^2+^ influx in MIN6 cells (A) GSIS of NC MIN6 cells under basal glucose (2.8 mM) or high glucose (16.7 mM) following various ultrasound doses (0–23.52 a.u.). NC cells showed minimal insulin-secretion changes across doses. (B) GSIS of MscL-expressing MIN6 cells, showing a dose-dependent increase in insulin secretion under high glucose, with maximal response at the 11.76 a.u. dose. (C) ΔGSIS (ratio of high-glucose to basal secretion). MscL-expressing cells displayed a significant enhancement in glucose responsiveness compared with NC cells. (D) Intracellular Ca^2+^ fluorescence intensity measured by Fluo-4 flow cytometry. Values were normalized to the NC group (set to 100%). MscL-expressing cells exhibited a robust dose-dependent increase in Ca^2+^ influx. Data are mean ± SD; *n* = 4 independent biological replicates per group (A–C) and *n* = 5 independent cultures per group (D). Statistical analysis used two-way ANOVA with Tukey’s post hoc test (A), one-way ANOVA with Dunnett’s post hoc test (B), two-way ANOVA (C), and two-way ANOVA on log_10_-transformed data (D). ∗*p* < 0.05. Exact test statistics (*F* values), degrees of freedom, and exact *p* values are provided in Table S2.

### Serum metabolomic profiling reveals systemic metabolic alterations within the acoustic window

Untargeted metabolomic profiling was performed to compare metabolic responses between LIPUS alone and LIPUS combined with MscL-G22S expression (LIPUS-MscL). Principal component analysis (PCA) revealed a clear separation between the two groups along PC1, which accounted for 26.62% of the total variance, indicating that MscL expression substantially reshaped the metabolic landscape elicited by ultrasound stimulation (Figure 8A). Hierarchical clustering analysis further demonstrated distinct metabolite expression patterns between the LIPUS and LIPUS-MscL groups (Figure 8B). Notably, the introduction of MscL-G22S was associated with coordinated alterations in metabolites involved in lipid signaling, sphingolipid metabolism, and inflammatory mediators, suggesting that mechanosensitive channel expression modulates ultrasound-triggered metabolic pathways rather than merely amplifying nonspecific ultrasound effects. Differential metabolite analysis identified 237 significantly altered metabolites between groups (variable importance in projection [VIP] > 1, *p* < 0.05), including 75 upregulated and 162 downregulated metabolites in the LIPUS-MscL group relative to LIPUS alone (Figure 8C). The volcano plot highlighted prominent changes in metabolites related to bioactive lipid metabolism, redox regulation, and amino acid metabolism, indicating a broad reprogramming of metabolic responses driven by MscL-dependent mechanotransduction.

**Figure 8:**
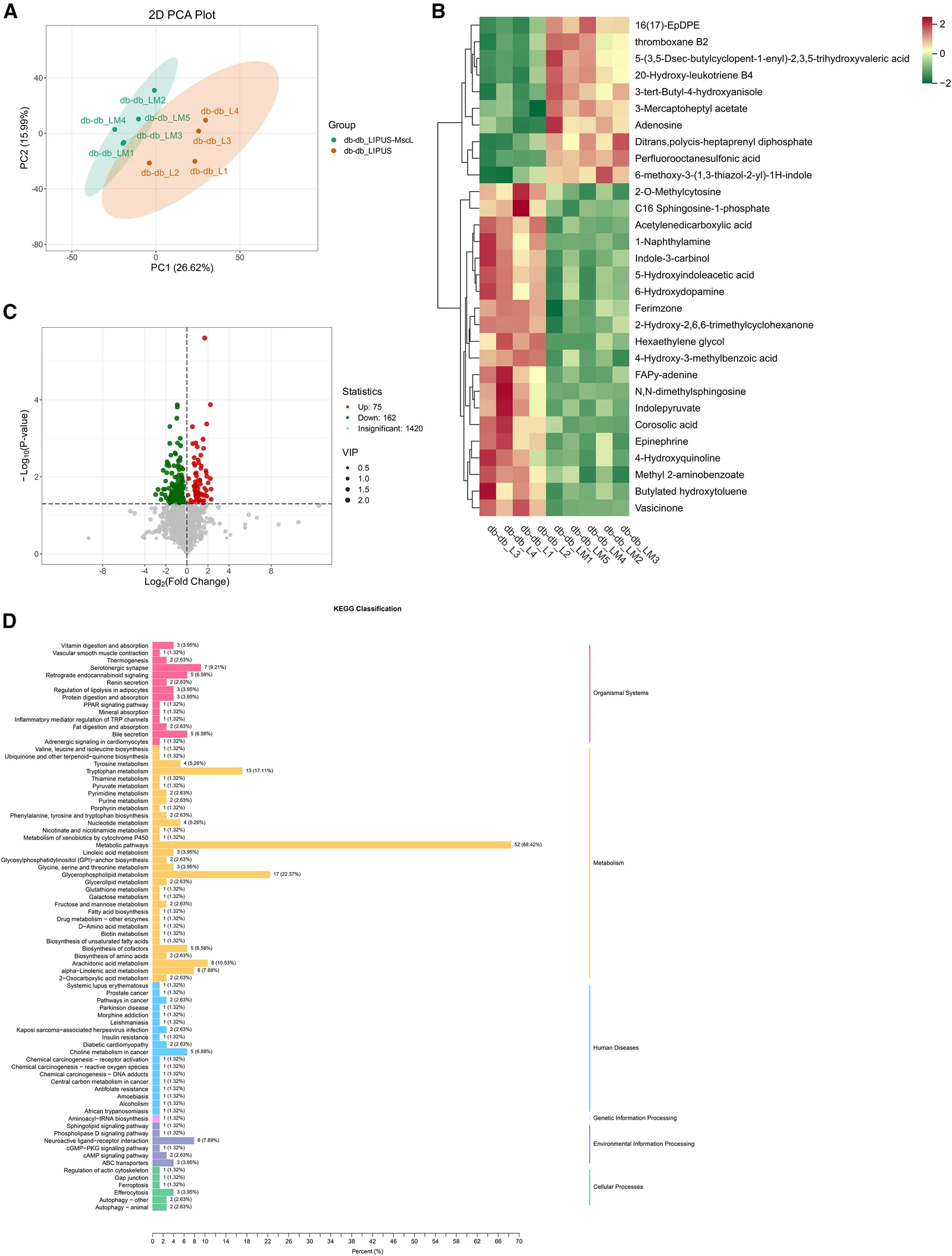
Metabolomic profiling of serum samples following LIPUS treatment (A) Principal component analysis (PCA) score plot showing clear separation between db/db mice treated with LIPUS alone and those receiving combined LIPUS-MscL activation (*n* = 4 mice per group). (B) Heatmap of differentially abundant metabolites between the two groups, with hierarchical clustering applied to both samples and metabolites. (C) Volcano plot illustrating significantly altered metabolites, defined by fold change and statistical significance thresholds (VIP > 1, Benjamini-Hochberg false discovery rate [FDR] < 0.05). (D) KEGG pathway classification of the significantly altered metabolites, highlighting pathways mainly associated with metabolic processes.

KEGG pathway enrichment analysis revealed that differentially regulated metabolites were significantly enriched in pathways associated with lipid metabolism, signal transduction, and endocrine and metabolic disease, including sphingolipid metabolism, arachidonic acid metabolism, insulin resistance, and inflammatory signaling pathways (Figure 8D). These pathway-level differences suggest that MscL expression biases ultrasound-induced metabolic responses toward pathways closely linked to glucose homeostasis and inflammatory regulation.

Importantly, to further evaluate whether MscL expression alone is sufficient to induce systemic metabolic remodeling, we performed an additional metabolomic comparison between the MscL-only group and the LIPUS-MscL group (Figure S4). As shown, MscL expression in the absence of ultrasound induced only limited and less coordinated metabolic alterations, whereas pronounced and pathway-level metabolic reprogramming was observed only under combined LIPUS-MscL stimulation.

Collectively, these findings suggest that MscL-G22S enhances and biases ultrasound-induced metabolic responses, rather than acting as an independent driver of metabolic remodeling, supporting the role of mechanosensitive channel activation in enabling more controlled and functionally relevant metabolic regulation *in vivo*.

## Discussion

This study systematically evaluated the feasibility of using LIPUS to activate the MSC, MscL-G22S, for non-invasive regulation of pancreatic function and glucose metabolism. Through *in vitro* mechanistic validation, AAV-mediated pancreatic expression, *in vivo* acoustic dose-response experiments, and serum metabolomic profiling, we demonstrate that the efficacy of sonogenetic metabolic modulation is governed by a defined acoustic dose window, rather than by ultrasound stimulation per se. This finding establishes acoustic dose optimization as a central determinant of therapeutic outcome and extends the application of sonogenetics beyond the nervous system to metabolic tissues.^18^^,^^19^

During the progression of type 2 diabetes, sedentary lifestyle and nutrient excess lead to chronic insulin resistance and sustained stress on pancreatic β cells, ultimately resulting in β cell dysfunction and hyperglycemia.^27^ In early disease stages, elevated glucose levels induce compensatory hyperinsulinemia,^28^ whereas progressive insulin resistance is accompanied by β cell dedifferentiation, functional decline, and loss of insulin secretory capacity.^29^ These pathophysiological features underscore the importance of developing non-invasive strategies capable of restoring or enhancing β cell function without chronic pharmacological intervention, particularly approaches that allow precise external control.

As a core effector in sonogenetics, the MSC, MscL possesses several favorable features—including structural simplicity, high membrane tension sensitivity, and a large pore diameter—that make it particularly suitable for ultrasound-mediated remote modulation. Previous studies have shown that activation of MscL enables ultrasonic control of neuronal activity,^25^ and that the gain-of-function mutant MscL-G22S lowers the mechanical activation threshold without inducing spontaneous channel opening, thereby enhancing ultrasound responsiveness.^30^^,^^31^ MscL-G22S has been reported to enhance cellular sensitivity to ultrasound stimulation *in vivo* and to induce robust Ca^2+^ influx and increased cellular activity under mechanical stress.^25^^,^^32^ In addition, ectopic expression of such mechanosensitive channels can confer high spatiotemporal precision to ultrasound stimulation,^33^ and sonogenetic strategies allow AAV-mediated targeting of ultrasound-sensitive proteins to specific cell populations.^17^ These properties align well with previous reports showing pressure-dependent activation of MscL-G22S.^23^^,^^24^^,^^25^^,^^26^ Consistently, our *in vitro* experiments demonstrated that LIPUS effectively opens MscL-G22S channels, triggers pronounced Ca^2+^ influx, and enhances insulin secretion under high-glucose conditions, in agreement with earlier observations that ultrasound can promote insulin release.^4^

*In vivo*, AAV-mediated expression of MscL-G22S successfully localized to pancreatic islets. Through systematic dose-response characterization, we identified a clear dose dependency in LIPUS-mediated activation of MscL-G22S, with glucose-lowering efficacy increasing with acoustic intensity and reaching an optimal range at approximately 3.5–5.9 a.u. (groups G2–G4). This pattern is consistent with known mechanical effects of ultrasound, such as modulation of membrane tension and permeability.^5^^,^^6^^,^^7^ These findings establish, to our knowledge, the first quantifiable *in vivo* acoustic activation window for a sonogenetic effector, providing a mechanistic explanation for inconsistent outcomes in prior ultrasound studies and offering a rational framework for parameter optimization. Notably, a modest glucose-lowering trend was observed in the LIPUS + MscL group under the conventional ultrasound parameters of the pilot study. However, this effect was limited and inconsistent across endpoints, indicating that MscL expression combined with suboptimal conventional stimulation is insufficient to drive robust metabolic regulation. Instead, these observations further support that effective metabolic modulation requires ultrasound-mediated activation of MscL within an appropriate acoustic dose range.

It is important to clarify that the identified acoustic dose window represents a biologically consistent, system-dependent response regime within the present experimental framework, arising from the combined effects of LIPUS and MscL, rather than a universally fixed parameter set. While our study demonstrates robust dose-dependent effects across multiple independent metabolic endpoints (including fasting blood glucose, glucose tolerance, insulin sensitivity, and insulin secretion) and consistent trends across *in vitro* and *in vivo* scales, we acknowledge that full cross-species or cross-model generalizability would require additional validation beyond the current proof-of-concept design. We also note that prior studies have extensively explored suitable ultrasound parameters for biological and metabolic modulation.^34^^,^^35^^,^^36^ At the systemic level, optimized LIPUS-MscL activation not only improved glucose tolerance and insulin sensitivity but also promoted favorable changes in lipid metabolism, reduced glycation burden, and induced broad metabolic pathway reprogramming. Metabolomic profiling revealed that the medium-dose group (G3) exhibited marked metabolic remodeling, including metabolic patterns suggestive of enhanced oxidative metabolism and altered carbohydrate, lipid, and amino acid metabolic pathways. These alterations are consistent with improved glucose homeostasis, enhanced insulin sensitivity, and broader metabolic adaptations induced by sonogenetic stimulation. These findings are consistent with previously reported time- and dose-dependent biological effects of ultrasound in diabetic tissues.^37^

In conclusion, this study establishes the acoustic dose window as a central principle for effective and safe sonogenetic modulation of glucose metabolism. Through systematic evaluation across cellular, physiological, and metabolomic levels, we identified a well-defined acoustic activation window that enables reproducible control of MscL-G22S-mediated responses. *In vitro*, ultrasound activation of MscL-G22S enhanced Ca^2+^ influx and GSIS, whereas in diabetic mice, it improved glucose homeostasis, alleviated insulin resistance, preserved islet architecture, and promoted favorable metabolic remodeling. These findings provide a foundation for the optimization and translational development of sonogenetics as a non-invasive strategy for metabolic regulation and support its potential application in the treatment of metabolic disorders such as type 2 diabetes.

### Limitations of the study

Despite these advances, several limitations remain. First, although the AAV/pan serotype used in this study exhibits broad tissue tropism and efficiently transduces the pancreas, it is not strictly β cell specific, and contributions from non-β cell populations cannot be fully excluded. Therefore, while our *in vitro* findings support a role for β cell modulation, the observed *in vivo* metabolic improvements likely reflect combined responses from multiple pancreatic cell populations and broader systemic regulatory mechanisms. Second, endogenous mechanosensitive channels (e.g., Piezo and TRP family members) may also contribute to ultrasound-induced biological responses; therefore, their involvement cannot be completely ruled out in the observed metabolic effects. Third, the present study uses relative a.u. rather than standardized physical parameters such as mechanical index or acoustic intensity, which limits direct cross-study comparability. In addition, ultrasound was applied at a fixed frequency (0.36 MHz) without spatial focusing, which may constrain the generalizability of the defined acoustic dose window. Fourth, in larger animal models and humans, significant ultrasound attenuation together with the deep anatomical location of the pancreas may pose challenges for efficient and spatially precise energy delivery. Fifth, while short-term metabolic improvements were clearly observed, the long-term durability and safety of repeated ultrasound stimulation require further investigation. Finally, although metabolomic profiling identified responsive metabolic pathways, the intracellular signaling cascades linking mechanosensitive channel activation to systemic metabolic remodeling remain to be fully elucidated. Collectively, these limitations indicate that the present study establishes a proof-of-concept sonogenetic strategy for metabolic regulation, while further optimization in acoustic standardization, targeting specificity, and delivery engineering will be required for future translational application. Future studies will focus on dissecting downstream signaling mechanisms, refining acoustic dose window parameters, and developing more tissue-specific or sensitivity-tuned MscL variants.

## Resource availability

### Lead contact

Requests for further information, resources, and reagents should be directed to and will be fulfilled by the lead contact, Yan Wang (wangyancq@cqmu.edu.cn).

### Materials availability

This study did not generate new unique reagents. The AAV vectors and plasmids used in this study were obtained from commercial vendors (BrainVTA [Wuhan] Co. Ltd. and GeneCopoeia, respectively) and are available for purchase from the respective companies. Requests for further information and resources may be directed to the lead contact.

### Data and code availability

The untargeted serum metabolomics data reported in this study have been deposited in the MassIVE repository (https://massive.ucsd.edu) (MassIVE: MSV000103292) and are publicly available as of the date of publication; the accession number is also listed in the key resources table. All other data reported in this paper will be shared by the lead contact upon request. This paper does not report original code. Any additional information required to reanalyze the data reported in this paper is available from the lead contact upon request.

## Acknowledgments

This work was supported by the Program for Chongqing Medical University Graduate Elite Talent Cultivation Project (BJRC202423) and the Science and Technology Research Program of Chongqing Municipal Education Commission (no. KJZD-M202200403). We thank the Catholic University of Louvain (Belgium) for kindly providing the MIN6 cell line.

## Author contributions

X.Z. conceived and designed the study and performed the experiments with assistance from Y.W. X.Z. and J.Z. drafted the manuscript with input from Y.W., W.M., and X.H. Y.C. and J.C. completed the detection of experimental indicators. X.Z. and W.M. analyzed and processed the data. Y.W. designed and supervised the entire project, reviewed the manuscript, and acquired funding. All authors read and approved the final manuscript.

## Declaration of interests

The authors declare no competing interests.

## STAR★Methods

### Key resources table

**Table:** 

REAGENT or RESOURCE	SOURCE	IDENTIFIER
**Antibodies**		

INS mouse McAb	Proteintech, Hubei, China	Cat# 66198-1-Ig; RRID: AB_2881591
Glucagon Rabbit PolyAb	Proteintech, Hubei, China	Cat# 15954-1-AP; RRID: AB_2878200
Alexa Fluor® 488-conjugated Goat Anti-Rabbit IgG (H + L) (1:1000)	Cell Signaling Technology, Shanghai, China	Cat# 4412S; RRID: AB_1904025
Alexa Fluor® 647-conjugated Goat Anti-Mouse IgG (H + L) (1:1000)	Cell Signaling Technology, Shanghai, China	Cat# 4410S; RRID: AB_1904023

**Chemicals, peptides, and recombinant proteins**		

Lipofectamine 3000	Invitrogen	Cat# L3000001
Fluo-4 AM calcium indicator	4A Biotech, Suzhou, China	Cat# FXP136-100
TRNzol Universal reagent	Tiangen Biotech (Beijing) Co., Ltd., Beijing, China.	Cat# DP424
Reverse transcription kit	TaKaRa Biotechnology Co., Ltd., Oita, Japan	Cat# RR037A
Opti-MEM	Gibco	Cat# 31985062
High-glucose DMEM	Gibco	Cat# 11965092
Fetal bovine serum (FBS)	Bio-Channel, Jiangsu, China	Cat# BC-SE-FBS01C
Penicillin–streptomycin	Beyotime Biotechnology, China	Cat# C0222
4% Paraformaldehyde	Guangzhou Saiguo Biotech Co., Ltd., China	Cat# BL539A
Ultrasound coupling agent (TM-100)	Tianjin Chengxin Medical Auxiliary Materials Factory	N/A
Recombinant human insulin	Novo Nordisk	N/A
D-glucose	Aladdin Biochemical, Shanghai, China	Cat# B1373171
Isoflurane	RWD Life Science, Shenzhen, China	N/A
H&E staining solution	Sangon Biotech, Shanghai, China	Cat# E607317

**Critical commercial assays**		

CCK-8 reagent	Beyotime Biotechnology, China	Cat# C0037
Mouse insulin ELISA kit	Jiangsu Jingmei Biotechnology, China	Cat# JM-02732M2
TC/TG/HDL-C/LDL-C assay kits	Nanjing Jiancheng Bioengineering Institute, China	Cat#: TC = A111-1-1, TG = A110-1-1, HDL-C = A112-1-1, LDL-C = A113-1-1
GSP colorimetric assay kit	Elabscience, Wuhan, China	Cat# E-BC-K069-M
Pierce BCA protein assay kit	Thermo Fisher Scientific	Cat# 23225

**Deposited data**		

Raw and processed untargeted serum metabolomics data	This study	MassIVE: MSV000103292; https://doi.org/10.25345/C5901ZW79

**Experimental models: Cell Lines**		

HEK293T	Shanghai Fuheng Biotechnology	Cat# FH0244
MIN6 (mouse pancreatic β-cell line)	Catholic University of Louvain, Belgium	RRID: CVCL_0431

**Experimental models: Organisms/strains**		

C57BKS-db/db mice (male, 8 weeks)	Changzhou Cavens Laboratory Animal Co., Ltd.	SCXK (Su) 2021–0013
C57BKS-db/m mice (male, 8 weeks)	Changzhou Cavens Laboratory Animal Co., Ltd.	SCXK (Su) 2021–0013

**Oligonucleotides**		

qPCR primers (MscL-G22S, 18S); sequences in Table S1	Sangon Biotech, Shanghai, China	N/A

**Recombinant DNA**		

rAAV-CBA-MscL-G22S-F2A-EGFP (pan serotype)	BrainVTA (Wuhan) Co., Ltd.	N/A
rAAV-CBA-EGFP (pan serotype, control vector)	BrainVTA (Wuhan) Co., Ltd.	N/A
MscL-G22S plasmid	GeneCopoeia	Cat# CS-GS3778-M56
Control plasmid	GeneCopoeia	Cat# EX-NEG-M56

**Software and algorithms**		

GraphPad Prism 8	GraphPad Software	https://www.graphpad.com; RRID: SCR_002798
ImageJ	NIH	https://imagej.nih.gov; RRID: SCR_003070
NIS-Elements Viewer 4.20	Nikon	RRID: SCR_014329
MetaboAnalyst 5.0	Xia Lab	https://www.metaboanalyst.ca; RRID: SCR_015539
SIMCA	Sartorius	RRID: SCR_014688

**Other**		

LIPUS device (USR-6)	Chongqing Ronghai Engineering Research Center of Ultrasound Medicine	N/A
Radiation force balance (UPM-DT-1)	Ohmic Instruments	N/A
Needle hydrophone (NHA-0400)	ONDA Corporation	N/A
Handheld glucometer (GA-3)	Sinocare, Changsha, China	N/A
Laser scanning confocal microscope (A1+R)	Nikon, Tokyo, Japan	N/A
Flow cytometer	Beckman Coulter, USA	N/A
Microplate reader	Tecan, Shanghai, China	N/A
qPCR instrument	Agilent Technologies, USA	N/A
Analytical balance (XS104)	Mettler Toledo, Switzerland	N/A

### Experimental model and study participant details

#### Animals

Male C57BKS-db/db mice (8 weeks old) and age-matched male C57BKS-db/m controls (SPF grade) were purchased from Changzhou Cavens Laboratory Animal Co., Ltd. (production license SCXK (Su) 2021–0013). Only male mice were used in this study; the potential influence of sex on the results was not assessed and is acknowledged as a limitation. All animals were housed in the SPF animal facility of the Laboratory Animal Center, Chongqing Medical University (license SYXK (Chongqing) 2022–0016) under controlled environmental conditions (22 ± 2 °C, 45 ± 5% humidity, 12 h light/dark cycle), with free access to food and water. All animal procedures were approved by the Institutional Animal Care and Use Committee (IACUC) of Chongqing Medical University (protocol number IACUC-CQMU-2025–01032) and were conducted in strict accordance with the standards of the Ethics Committee of the Animal Experiment Center of Chongqing Medical University, conforming to all relevant regulatory standards for animal research.

#### Cell lines

HEK293T cells were purchased from Shanghai Fuheng Biotechnology Co., Ltd. (catalog no. FH0244). Cells were maintained in high-glucose DMEM (Gibco) supplemented with 10% FBS (Bio-Channel, Jiangsu, China) and 1% penicillin–streptomycin (Beyotime, China) at 37 °C in 5% CO_2_. 293T cells were used for plasmid transfection efficiency validation and served as a parallel model for assessing MscL-G22S cytotoxicity and ultrasound tolerance. Cells were authenticated by short tandem repeat (STR) profiling by the vendor (Shanghai Fuheng Biotechnology, Shanghai, China) and tested negative for mycoplasma contamination in the vendor’s pre-shipment quality inspection.

MIN6 mouse pancreatic β-cells were kindly provided by the Catholic University of Louvain (Belgium). Cells were cultured in high-glucose DMEM (Gibco) containing 15% FBS, 1% penicillin–streptomycin, and 50 μM β-mercaptoethanol at 37 °C with 5% CO_2_. MIN6 cells were authenticated by short tandem repeat (STR) profiling (Shanghai Fuheng Biotechnology, Shanghai, China), which confirmed mouse-cell origin with no cross-species contamination, and tested negative for mycoplasma contamination by quantitative PCR.

### Method details

#### AAV vector construction and delivery

The rAAV-CBA-MscL-G22S-F2A-EGFP vector and the rAAV-CBA-EGFP control vector were produced by BrainVTA (Wuhan) Co., Ltd. and packaged in the vendor-designated pan capsid serotype. Final genome titers were ≥5 × 10^12^ vg/mL (qPCR). Mice received 5 × 10^11^ vg via tail-vein injection.

#### LIPUS treatment (pilot study)

A preliminary *in vivo* experiment was conducted to test the biological response to conventional LIPUS parameters. LIPUS was applied using the USR-6 ultrasound device (Chongqing Ronghai Engineering Research Center of Ultrasound Medicine, China). Acoustic output was calibrated in degassed water under controlled temperature conditions using a radiation force balance (UPM-DT-1, Ohmic Instruments, USA) and validated with a calibrated needle hydrophone (NHA-0400, ONDA Corporation, USA). Acoustic pressure measurements were performed at the focal region of the transducer under free-field conditions, and spatial-peak temporal-average intensity (ISPTA) was derived accordingly. Each measurement was repeated across multiple trials to ensure reproducibility, and variability was recorded as mean ± SD. After establishing the diabetic model, mice underwent abdominal depilation and were given daily LIPUS irradiation directed to the pancreatic region for 10 consecutive days. A 25-mm probe delivered ultrasound with the following fixed parameters: 1.176 a.u. [200 mW/cm^2^, 10 min/day, 0.36 MHz, 1 kHz pulse repetition frequency (PRF), 20% duty cycle]. Sham mice received probe placement without energy output. In mice, the pancreas is located in the left upper abdominal quadrant, extending from the duodenum to the spleen, mainly below the left costal margin. A sterile marker was used to delineate the projection area: a rectangular region (longitudinal axis 10–15 mm, transverse axis 6–10 mm) was marked approximately 1–2 cm below the left costal margin and lateral to the ventral midline, with the xiphoid process as the reference point. This area was adjusted slightly leftward to ensure coverage of the head, body, and tail of the pancreas, and verified in pilot animals by anatomical dissection. The transducer was placed in the irradiation area and contacted with the skin through a coupling agent (Jinya TM-100, Tianjin, China), and a metal stand was used to stabilize the probe during treatment. This pilot study was used to assess feasibility and guide optimization of acoustic parameters for the dose-dependent study.

#### *In vivo* LIPUS treatment (dose-dependent study)

Based on pilot findings, a dose-dependent LIPUS protocol was implemented. Mice were restrained on a standard mouse board without anesthesia to avoid metabolic interference. Ultrasound coupling gel was applied, and LIPUS was targeted to the pancreatic region. Treatments followed the acoustic parameters listed in Table 1, including graded intensities, duty cycles, and daily irradiation durations for 10 days. Parameters were selected based on preliminary dose–response experiments to cover a physiologically responsive range without causing tissue damage. Mice were monitored throughout to ensure minimal stress. Acoustic dose is expressed in arbitrary units (a.u.), defined as the cumulative delivered ultrasound energy calculated from acoustic intensity, effective transducer area, duty cycle, and irradiation duration; in the *in vivo* study, 1 a.u. corresponds to 1 kJ of calculated energy (Table 1), and in the *in vitro* study, 1 a.u. corresponds to 1 J of calculated energy (Table 2). For control purposes, a Mock group was included, in which mice received tail-vein injection of an empty AAV vector (lacking the MscL-G22S transgene) to control for potential non-specific effects of AAV administration. Mice in the Mock group underwent identical restraint, gel application, and pancreatic-region targeting procedures as the treatment groups, but were not exposed to active LIPUS stimulation (sham treatment). All groups were housed and monitored under identical conditions throughout the study. A second round of LIPUS treatment was subsequently administered using the same group-specific acoustic parameters (Table 1), beginning at 14 weeks of age (i.e., 2 weeks after the completion of the first round) and likewise comprising 10 consecutive daily sessions. Blood glucose was monitored immediately after each treatment round and at defined intervals thereafter until the study endpoint (20 weeks of age).

#### Body weight and blood glucose monitoring

Body weight and random blood glucose levels were monitored once weekly throughout the treatment period. Blood glucose was measured from the tail vein using a handheld glucometer (GA-3, Sinocare, China). Fasting blood glucose (FBG) was assessed after a 6-h fast at baseline and at the end of the LIPUS intervention.

#### Food and water intake

Food and water intake were recorded every 2 days. Consumption was calculated as the difference between the pre-measured food/water amount and the remaining amount at each time point. Intake was normalized to the number of mice per cage.

#### Glucose tolerance test (GTT) and insulin tolerance test (ITT)

For the glucose tolerance test (GTT), mice were fasted for 16 h and then intraperitoneally injected with a 20% D-glucose solution (2 g/kg body weight). For the insulin tolerance test (ITT), mice fasted for 6 h were injected intraperitoneally with human insulin (0.75 U/kg body weight). Blood glucose levels were measured from the tail vein at 0, 15, 30, 60, 90, and 120 min post-injection using a glucometer. The area under the curve (AUC) was calculated for each test.

#### Serum lipid profile

Serum lipid measurements were performed both before and after the LIPUS treatment. Mice were fasted for 6 h, and blood samples were collected from the orbital sinus at baseline and at the end of the treatment period. Serum was separated by centrifugation at 3,000 rpm for 10 min. Levels of total cholesterol (TC), triglycerides (TG), low-density lipoprotein cholesterol (LDL-C), and high-density lipoprotein cholesterol (HDL-C) were quantified using commercial assay kits from Nanjing Jiancheng Bioengineering Institute (Nanjing, China), following the manufacturer’s instructions. Absorbance was measured using a microplate reader, and lipid parameters were calculated accordingly.

#### Glycosylated serum protein (GSP) measurement

Serum GSP levels were measured at 16 and 20 weeks of age using a commercial colorimetric assay kit (Elabscience, Wuhan, China) according to the manufacturer’s instructions. Absorbance was measured with a microplate reader, and GSP concentrations were calculated accordingly.

#### Tissue collection and eWAT weighing

At the study endpoint (20 weeks of age), mice were euthanized under deep isoflurane anesthesia, and the epididymal white adipose tissue (eWAT) was dissected and weighed; the eWAT-to-body-weight ratio was calculated for each animal. Pancreatic tissue and major organs (heart, liver, spleen, lung, and kidney) were harvested for histological analysis as described below.

#### Hematoxylin and eosin (H&E) staining of tissue samples

Pancreatic tissue and other harvested organs were rinsed with normal saline and fixed in 4% paraformaldehyde (Guangzhou Saiguo Biotech Co., Ltd., China) for 24 h. After fixation, samples were processed through routine dehydration, paraffin embedding, and sectioning at 5 μm thickness. H&E staining was performed following standard procedures. Histopathological features were examined under a light microscope (Olympus, Tokyo, Japan) in five randomly selected fields per section.

#### Enzyme-linked immunosorbent assay (ELISA)

The concentrations of insulin in serum were determined using specific commercial ELISA kits (Jiangsu Jingmei Biotechnology Co., China) according to the manufacturer’s instructions. The absorbance was measured at 450 nm using a microplate reader, and insulin concentrations were interpolated from standard curves. HOMA-IR was calculated using the following formula: HOMA-IR = [fasting glucose (mmol/L) × fasting insulin (μU/mL)]/22.5.

#### Immunofluorescence (IF)

Paraffin-embedded pancreatic sections were deparaffinized, rehydrated, and subjected to antigen retrieval in citrate buffer (pH 6.0). After permeabilization and blocking, sections were incubated overnight at 4 °C with primary antibodies against insulin (1:200; Proteintech, Hubei, China) and glucagon (1:200; Proteintech, Hubei, China). After washing, FITC-conjugated (1:200) and DyLight 649-conjugated (1:500) goat anti-rabbit IgG secondary antibodies were applied for 1 h , and nuclei were counterstained with DAPI. Images were acquired using a Nikon A1+R laser scanning confocal microscope (Nikon, Tokyo, Japan) with 488-nm and 640-nm lasers, and fluorescence intensity was quantified using ImageJ in five randomly selected fields. Image acquisition was performed with NIS-Elements Viewer 4.20 software (Nikon).

#### Plasmid transfection

293T and MIN6 cells were seeded into 35-mm dishes one day before transfection. Cells were transfected using Lipofectamine 3000 (Invitrogen) according to the manufacturer’s instructions. For each dish, 2.5 μg plasmid DNA (MscL-G22S plasmid CS-GS3778-M56 or control plasmid EX-NEG-M56, GeneCopoeia) was mixed with 5 μL P3000 and 5 μL Lipofectamine 3000 in Opti-MEM to form transfection complexes. The mixture was added dropwise to cells and incubated for 24–48 h before subsequent assays, including CCK-8 viability analysis, Ca^2+^ measurement, and GSIS.

#### *In vitro* ultrasound stimulation

LIPUS was applied to 293T and MIN6 cells using a 0.36-MHz transducer. Treatment frequency, duration, and acoustic dose levels were administered according to the predefined protocol shown in Table 2 to assess dose-dependent cellular responses. After stimulation, cells were immediately subjected to calcium imaging, insulin secretion assays, and viability analysis.

#### CCK-8 cell viability assay

Cell viability was assessed using the CCK-8 assay (Beyotime, China). 293T and MIN6 cells were seeded into 96-well plates and allowed to attach overnight. After plasmid transfection (MscL-G22S or control) and/or LIPUS treatment, 10 μL of CCK-8 solution was added to each well and incubated for 2 h at 37 °C. Absorbance at 450 nm was measured using a microplate reader, and viability was calculated according to the manufacturer’s instructions.

#### Glucose-stimulated insulin secretion (GSIS) assay

MIN6 cells were starved at 37 °C for 1 h in Krebs–Ringer bicarbonate (KRB) buffer containing 2.8 mM glucose, followed by incubation at 37 °C for 1 h in KRB buffer supplemented with either 2.8 or 16.7 mM glucose. Insulin levels in the supernatants of cultured cells or islets, as well as in cultured cells (lysed in ice-cold HCl–ethanol), were quantified by ELISA. For islets, the levels of secreted insulin were normalized to the total protein content of the islets, which was determined using a Pierce BCA protein assay kit (Thermo Fisher Scientific).

#### Calcium imaging

Intracellular Ca^2+^ levels were measured using Fluo-4 AM. MIN6 cells were incubated with Fluo-4 AM (5 μM) for 30 min at 37 °C, washed, and resuspended in pre-warmed buffer. Cells were then subjected to LIPUS stimulation, and fluorescence intensity was immediately quantified using a flow cytometer (FITC channel). Mean fluorescence intensity (MFI) was used to indicate intracellular Ca^2+^ levels.

#### Quantitative real-time PCR (qRT-PCR)

Total RNA was extracted from pancreatic tissues, other harvested tissues, and cultured 293T or MIN6 cells using TRIzol reagent according to the manufacturer’s protocol. RNA concentration and purity were determined using a NanoDrop spectrophotometer, and samples with an A260/A280 ratio between 1.8 and 2.0 were used for subsequent analysis. RNA was reverse transcribed into cDNA using a reverse transcription kit (TaKaRa Biotechnology Co., Ltd., Oita Prefecture, Japan), and quantitative real-time PCR was performed on a qRT-PCR instrument (Agilent Technologies Co., Ltd., CA). The PCR cycling conditions were as follows: initial denaturation at 95 °C for 30 s, followed by 40 cycles of 95 °C for 5 s and 60 °C for 30 s. Gene expression levels were normalized to the housekeeping gene 18S and calculated using the comparative 2^∧^(-ΔΔCt) method. All primer sequences used in this study are provided in Table S1.

#### Untargeted metabolomic analysis

Untargeted metabolomic profiling was performed on serum samples collected at week 20 (end of study) to characterize the systemic metabolic effects of the interventions. For sample preparation, 50 μL of serum was mixed with pre-chilled methanol (4:1, v/v) containing internal standards, vortexed, incubated on ice, and centrifuged at 12,000 × g for 10 min at 4 °C. The supernatant was collected for LC-MS analysis. Chromatographic separation was performed using a UHPLC system coupled to a high-resolution mass spectrometer operated in both positive and negative electrospray ionization modes. Quality control (QC) samples were prepared by pooling aliquots of all serum samples and injected periodically to monitor system stability. The number of biological replicates in each group was *n* = 4.

Raw data were processed using vendor-specific software for peak detection, alignment, and normalization. After data preprocessing, multivariate analyses including principal component analysis (PCA) and orthogonal partial least squares discriminant analysis (OPLS-DA) were conducted using SIMCA or MetaboAnalyst 5.0. Differential metabolites were identified based on a combination of variable importance in projection (VIP >1), and adjusted *p* values (FDR <0.05). Multiple testing correction was performed using the Benjamini–Hochberg false discovery rate (FDR) method. Metabolic pathway enrichment and impact analyses were performed with the KEGG and MetaboAnalyst databases.

### Quantification and statistical analysis

All statistical analyses were performed using GraphPad Prism 8 (GraphPad Software Inc., San Diego, CA). Data are reported as mean ± SD unless otherwise stated. Comparisons between two groups were performed using unpaired two-tailed Student’s t tests; before-versus-after comparisons within the same animals were performed using paired two-tailed Student’s t tests or two-tailed Wilcoxon matched-pairs signed rank tests, as specified in Table S2. For experiments involving more than two groups, one-way ANOVA with Tukey’s multiple comparisons test was applied; when variances were unequal, Welch’s one-way ANOVA followed by the Games–Howell post hoc test was used. For comparisons of multiple dose groups against a single control group, one-way ANOVA followed by Dunnett’s multiple comparisons test was used. For analyses involving both treatment group and time factors, two-way repeated-measures ANOVA with Geisser–Greenhouse sphericity correction followed by Tukey’s or Dunnett’s post hoc test was used, as specified in the figure legends and Table S2. When missing values precluded repeated-measures ANOVA, a mixed-effects model (REML) with the Geisser–Greenhouse correction was fitted instead. Flow cytometric Ca^2+^ data (Fluo-4 MFI) were log_10_-transformed before statistical analysis. For the acoustic intensity dose–response experiments, statistical comparisons across multiple intensity groups were performed using one-way ANOVA with Tukey’s post hoc tests. Linear trend and polynomial trend analyses were used to assess dose-dependent relationships. LOESS smoothing was applied for graphical visualization of dose–response curves, but not for statistical inference. qRT-PCR data were analyzed using one-way ANOVA with Tukey’s post hoc test, or Welch’s one-way ANOVA with Games–Howell post hoc test when variances were unequal. Correlation analyses were performed using Pearson’s correlation coefficients. Metabolomic datasets were analyzed using PCA, OPLS-DA, VIP scoring, and pathway enrichment analysis with MetaboAnalyst 5.0. A *p* value <0.05 was considered statistically significant. In the figure legends, n represents the number of mice per group (*in vivo* experiments) or independent biological replicates (*in vitro* experiments), and asterisks are defined in each legend (∗*p* < 0.05). A complete summary of all statistical tests—including test statistics (F and t values), degrees of freedom, exact *p* values, and the number of biological units for each figure panel—is provided in Table S2.

## Supplemental information

Document S1. Figures S1–S4 and Table S1

Table S2. Complete statistical summary for all figures, including test statistics (*F* and *t* values), degrees of freedom, exact *p* values, and the number of biological units
