# Supplementary material for: Non-invasive glycemic control leveraging ultrasound dose window activation of MscL in diabetic mice
Source: iScience. 2026 Oct 19;29(11):117962. doi: 10.1016/j.isci.2026.117962 (PMC13697812; doi:10.1016/j.isci.2026.117962)
Supplement: Supplementary file 1 [file mmc1.pdf]

iScience, Volume *29*

Supplemental information

Non-invasive glycemic control leveraging ultrasound dose window activation of MscL in diabetic mice

Xinyi Zhang, Jun Zhan, Wenjin Mao, Xinbin Han, Yu Chen, Jianghao Chen, and Yan Wang

Figure S1. Widespread AAV-mediated MscL-G22S expression across pancreatic islets.

Low-magnification imaging of pancreatic sections showing the overall distribution of AAV-delivered MscL-G22S-EGFP (green). The panel includes separate channels for nuclear staining (DAPI, blue), insulin-positive beta-cells (INS, red), and the merged view. Multiple pancreatic islets are visible, with MscL-G22S expression detected in insulin-positive regions, confirming widespread transduction of islet cells across the pancreas. Scale bar, 100 µm.

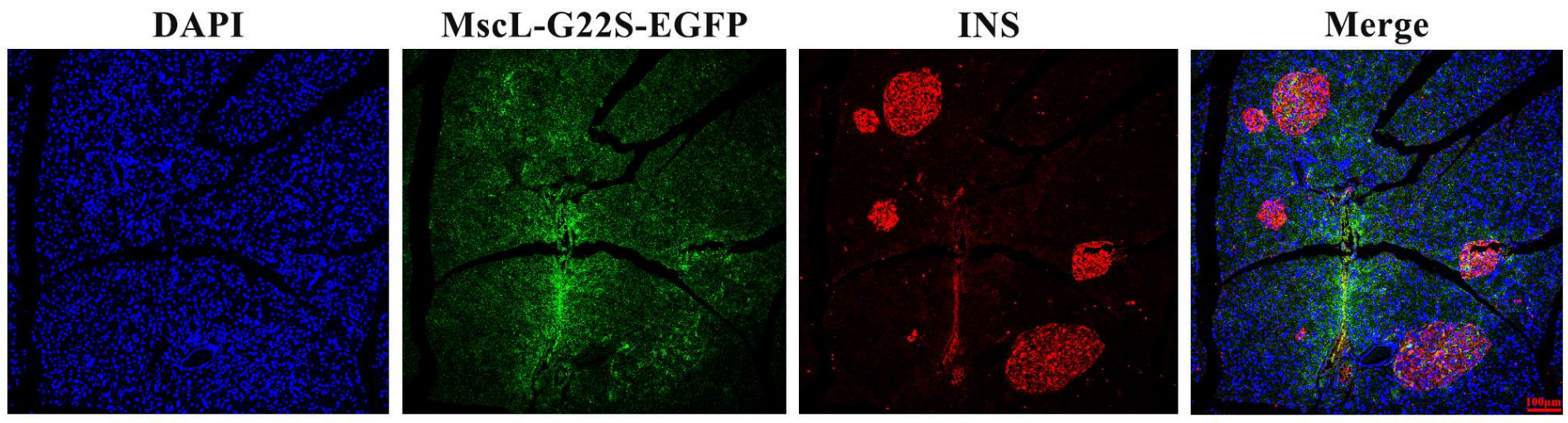

Figure S2. Glucose tolerance and insulin sensitivity under different ultrasound treatment conditions.

(A) GTT blood glucose levels at 0, 30, 60, 90, and 120 min after glucose administration in db/m control, db/db control, and dose groups G1-G5. (B) GTT area under the curve (AUC); comparison between db/db control and G3. (C) ITT blood glucose levels at indicated time points. (D) ITT AUC; comparison between db/db control and G3. Data are represented as mean +/- SD; n = 4 mice per group. Statistical significance in (A) and (C) was determined by two-way repeated-measures ANOVA with Geisser-Greenhouse correction followed by Dunnett's post hoc test versus the db/db control group within each time point; significance in (B) and (D) was determined by unpaired two-tailed Student's t test (db/db control vs. G3). *$p < 0.05$. Exact test statistics, degrees of freedom, and exact p values are provided in Table S2.

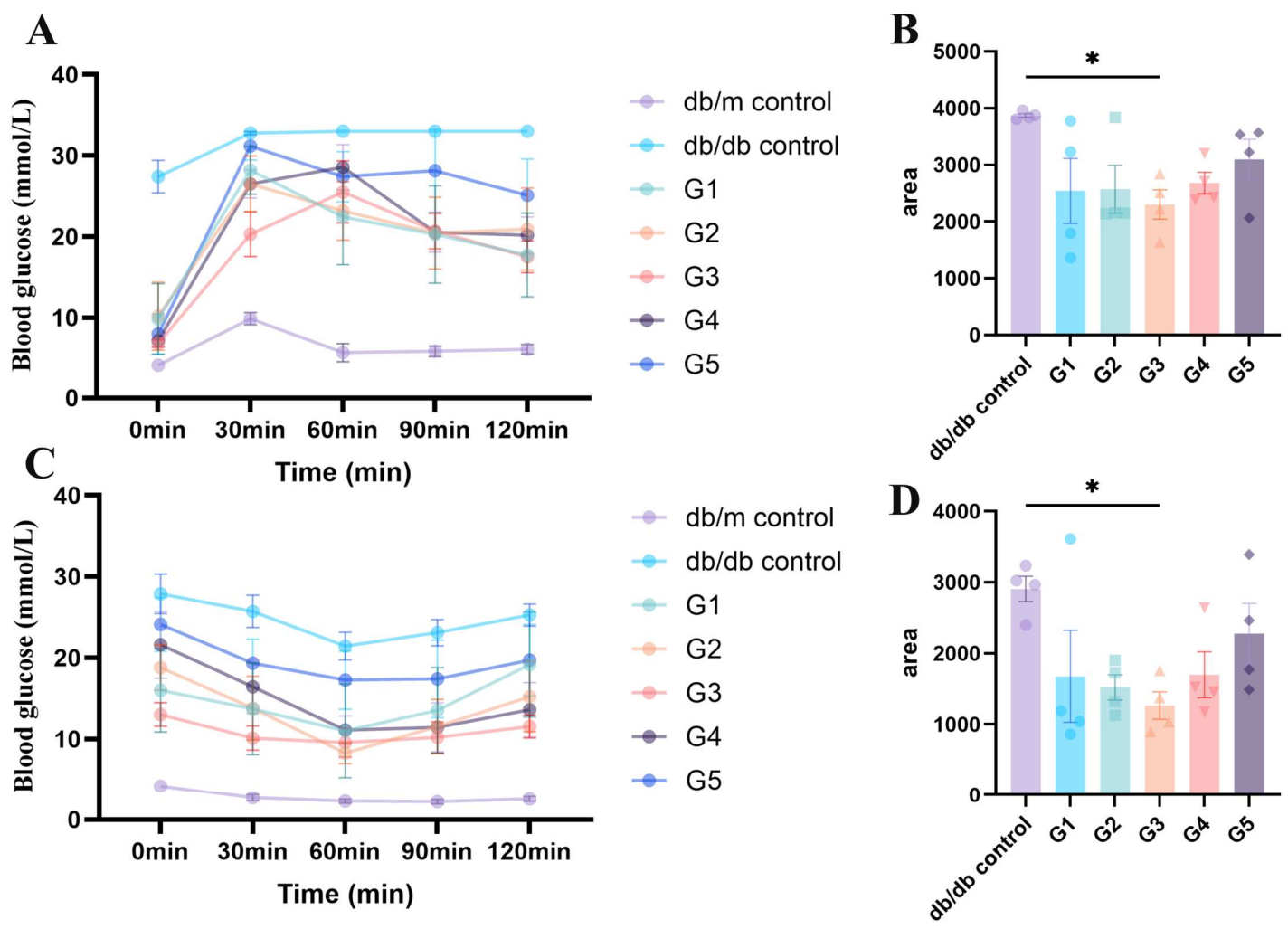

Figure S3. Representative H&E staining of major organs after ultrasound treatment.

Representative H&E-stained sections of heart, liver, spleen, lung, and kidney from db/m and db/db mice subjected to control, LIPUS, or LIPUS+MscL treatment. No gross histological abnormalities were detected across the examined organs. Scale bars: 100 μm (heart) and 200 μm (liver, spleen, lung, and kidney).

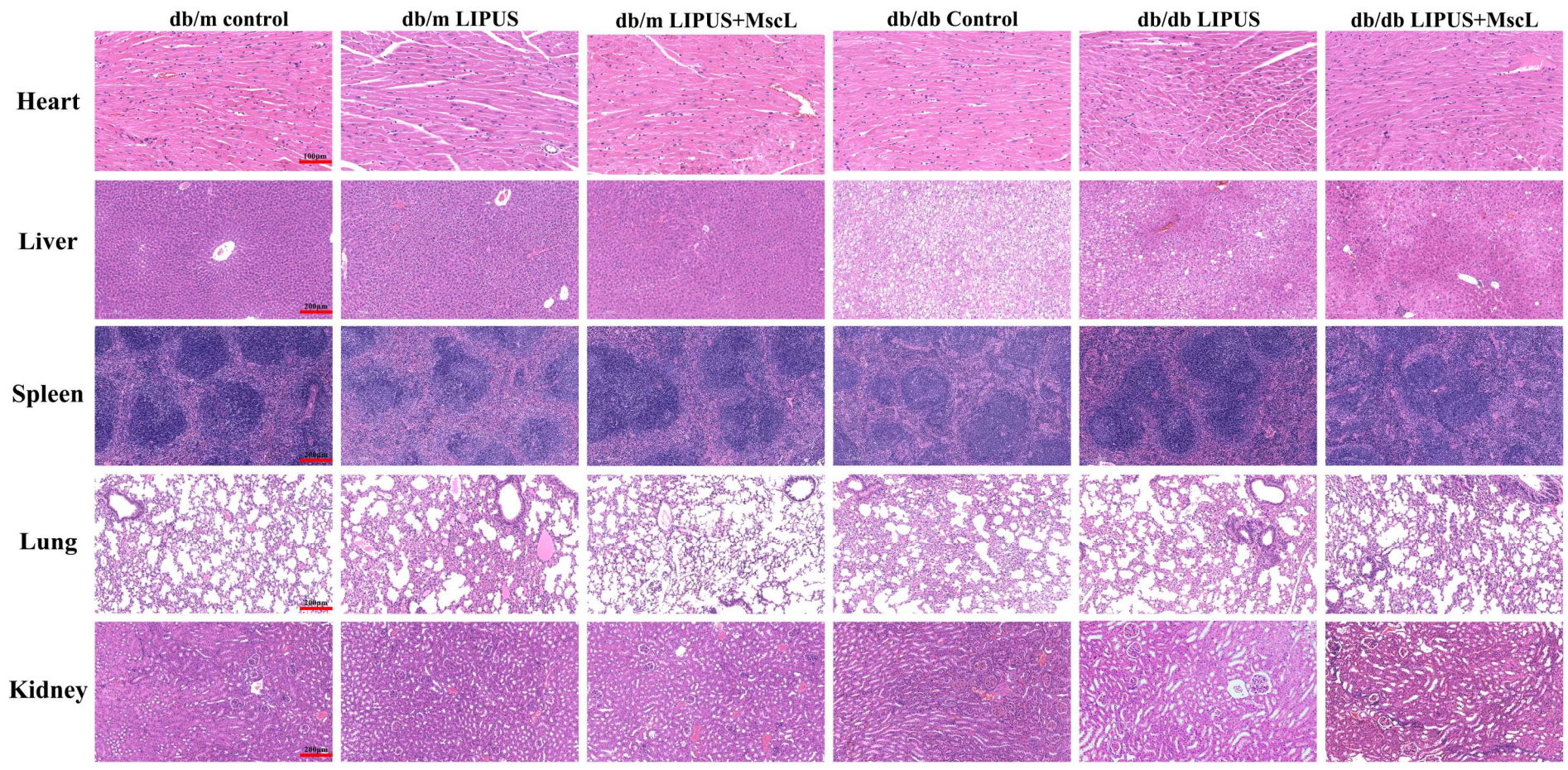

Figure S4. Serum metabolomic comparison between MscL expression alone and combined LIPUS-MscL activation.

(A) Principal component analysis (PCA) score plot showing clear separation between db/db mice with MscL-G22S alone and those receiving combined LIPUS-MscL activation. (B) Heatmap of differentially abundant metabolites between the two groups, with hierarchical clustering applied to both samples and metabolites. (C) Volcano plot illustrating significantly altered metabolites, defined by fold change and statistical significance thresholds. (D) KEGG pathway classification of the significantly altered metabolites, highlighting pathways mainly associated with metabolic processes.

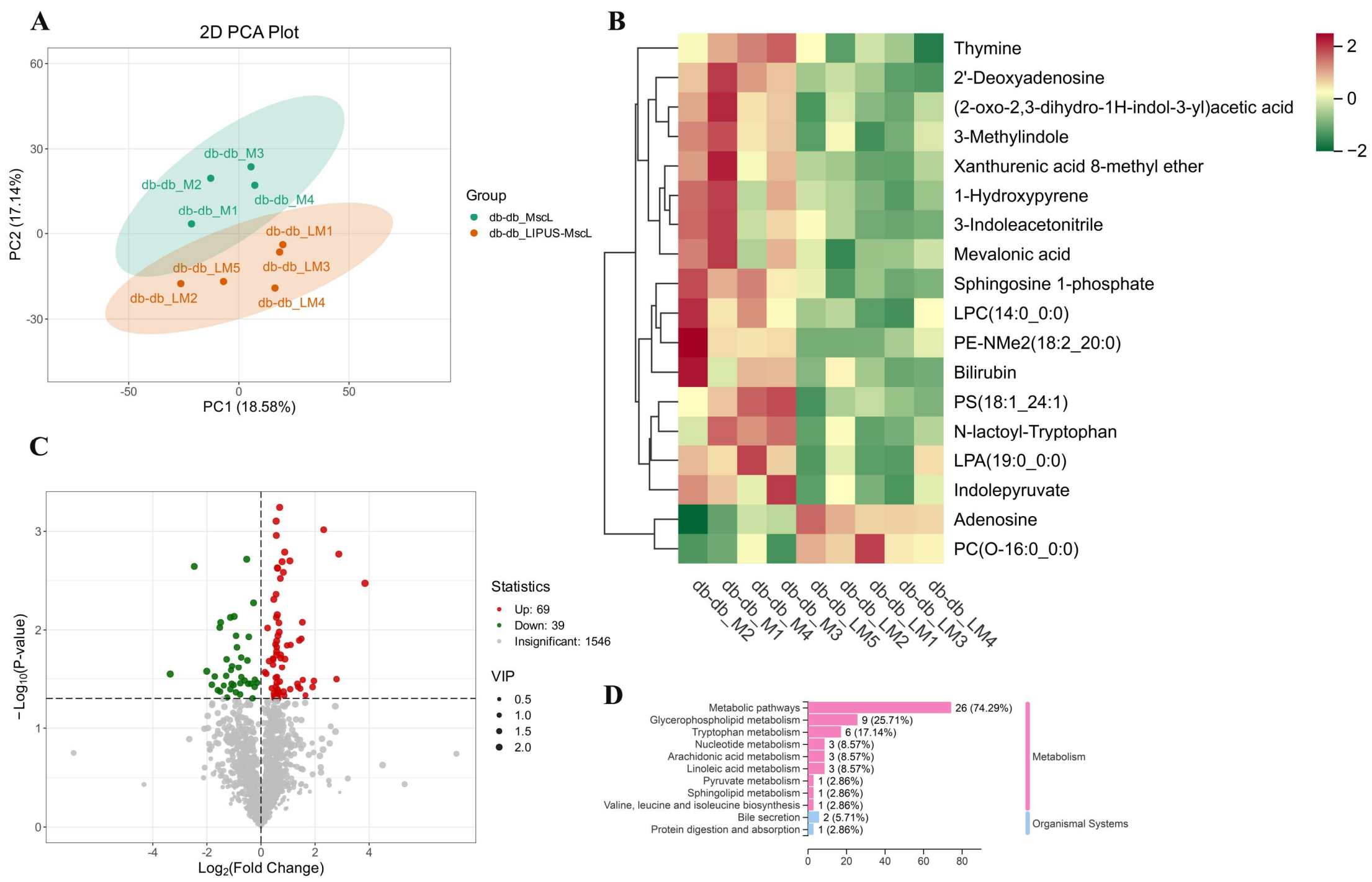

Table S1. Primer sequences targeting MscL-G22S and 18S used for qRT-PCR analysis.

Gene	Primer sequence
MscL-G22S	F: 5'-AAAACGTCTTTGATTTTCTGATTGTGGC-3'<br>R: 5'-CTGTCAGTAATACTTCTTCTTTAGTTGGTGC-3'
18S	F: 5'-CGGCTACCACATCCAAGGAA-3'<br>R: 5'-GCTGGAATTACCGCGGCT-3'

F: Forward primer; R: Reverse primer
